# Association of prenatal counselling and immediate postnatal support with early initiation of breastfeeding in Uttar Pradesh, India

**DOI:** 10.1186/s13006-021-00372-6

**Published:** 2021-03-16

**Authors:** Vasanthakumar Namasivayam, Bidyadhar Dehury, Ravi Prakash, Marissa Becker, Lisa Avery, Deepa Sankaran, B. M. Ramesh, James Blanchard, Pankaj Kumar, John Anthony, Manish Kumar, Ties Boerma, Shajy Isac

**Affiliations:** 1 Institute of Global Public Health, University of Manitoba, Winnipeg, Canada; 2 India Health Action Trust, New Delhi/Lucknow, India; 3 Government of Uttar Pradesh, Lucknow, India

**Keywords:** Early initiation of breastfeeding, Prenatal counselling on breastfeeding, Postnatal breastfeeding support, Uttar Pradesh, India, Uttar Pradesh technical support unit

## Abstract

**Background:**

Timely initiation of breastfeeding, also known as early initiation of breastfeeding, is a well-recognized life-saving intervention to reduce neonatal mortality. However, only one quarter of newborns in Uttar Pradesh, India were breastfed in the first hour of life. This paper aims to understand the association of community-based prenatal counselling and postnatal support at place of delivery with early initiation of breastfeeding in Uttar Pradesh, India.

**Methods:**

Data from a cross-sectional survey of 9124 eligible women (who had a live birth in 59 days preceding the survey) conducted in 25 districts of Uttar Pradesh, India, in 2018, were used. Simple random sampling was used to randomly select 40 Community Development Blocks (sub district administrative units) in 25 districts. The Primary Sampling Units (PSUs), health service delivery unit for frontline workers, were selected randomly from a linelisting of PSUs in each selected Community Development Block. Bivariate and multivariate logistic regression analyses were performed to assess the association of prenatal counselling and postnatal support on early initiation of breastfeeding in public, private and home deliveries.

**Results:**

Overall 48.1% of mothers initiated breastfeeding within an hour, with major variation by place of delivery (61.2% public, 23.6% private and 32.6% home). The adjusted odds ratio (aOR) of early initiation of breastfeeding was highest among mothers who received both counselling and support (aOR 2.67; 95% CI 2.30, 3.11), followed by those who received only support (aOR 1.99; 95% CI 1.73, 2.28), and only counselling (aOR 1.40; 95% CI 1.18, 1.67) compared to mothers who received none. The odds of early initiation of breastfeeding was highest among mothers who received both prenatal counselling and postnatal support irrespective of delivery at public health facilities (aOR 2.49; 95% CI 2.07, 3.01), private health facilities (aOR 3.50; 95% CI 2.25, 5.44), or home (aOR 2.84; 95% CI 2.02, 3.98).

**Conclusions:**

A significant association of prenatal counselling and postnatal support immediately after birth on improving early initiation of breastfeeding, irrespective of place of delivery, indicates the importance of enhancing coverage of both the interventions through community and facility-based programs in Uttar Pradesh.

**Supplementary Information:**

The online version contains supplementary material available at 10.1186/s13006-021-00372-6.

## Background

Uttar Pradesh (UP), the most populous state in India, requires a substantial reduction in Neonatal Mortality Rate (NMR) to attain the Sustainable Development Goal of 12 neonatal deaths per 1000 live births by 2030. Despite a reduction in the NMR of UP from 45 in 2005 to 32 in 2018, it remains higher than the national average (23 per 1000 live births) [1]. Early initiation of breastfeeding, defined as the initiation of breastfeeding within 1 h after delivery [2], is a well-recognized life-saving intervention to reduce NMR. It is estimated that early initiation of breastfeeding could avert approximately 22% of neonatal deaths globally [3]. The risk of neonatal death increases by 1.3 times when the initiation is delayed between 2 and 23 h [4–6] and it doubles when the initiation is delayed beyond 24 h [7]. Moreover, early initiation of breastfeeding has several other health benefits for both mothers and newborns as it stimulates breast milk production, reduces postpartum haemorrhage, protects newborns against diarrhoea and respiratory infections, improves mother-child bonding, and promotes the establishment of continued and exclusive breastfeeding [8, 9].

In 2015–16, only half of the newborns globally and 42% in India received early initiation of breastfeeding (8, 10). The early initiation of breastfeeding rate was much lower (25%) in UP during the same period [10]. Mothers need adequate and appropriate information on breastfeeding, and postnatal support on positioning, timing, and attachment to initiate breastfeeding within an hour [11–15]. A systematic review from South Asia identified lack of availability of correct information, as well as misconceptions about breastfeeding, as important barriers to early initiation of breastfeeding [16]. Moreover, physical support immediately after birth on positioning for breastfeeding is one of the ten important steps for successful breastfeeding as described by the World Health Organization (WHO) and United Nations Children Fund (UNICEF) [11].

Although studies have documented the factors associated with early initiation of breastfeeding such as mothers’ characteristics, place of residence (rural/urban), frequency of prenatal visits, access to healthcare facilities for delivery, mode of delivery and prelacteal feeding practice [14, 17–22], evidence on the association of prenatal counselling on breastfeeding as well as postnatal support on early initiation of breastfeeding remains limited in India as well as in UP. We aim to fill this information gap by analysing the differentials in early initiation of breastfeeding between mothers who received both prenatal counselling on breastfeeding and the postnatal support for breastfeeding, mothers who received either, compared to those who received none using representative data from the 25 High Priority Districts (HPD) of UP, India. Therefore, the focus of this paper is to understand the possible multiplicative effect and the difference in magnitude of association when prenatal counselling and postnatal support considered as a separate interventions versus a combined intervention. The programmatic insights generated through this study will help inform policymakers in prioritizing interventions to increase early initiation of breastfeeding rates.

## Methods

### Study setting

To ensure equity in healthcare and improve health outcomes, the Government of India and the Government of UP prioritized 25 districts based on poor health indicators and termed them as HPDs [23]. These 25 HPDs have a total population of 69.6 million, accounting for 35% of the state population [24]. The University of Manitoba and India Health Action Trust established the Uttar Pradesh Technical Support Unit (UPTSU) to provide techno-managerial support for improving maternal, neonatal and child health outcomes in these high priority districts. Within the 25 HPDs, 100 Community Development (CD) blocks equivalent to sub-districts, from 294 CD blocks were selected as focused CD blocks for the intensification and as learning labs.

Frontline workers (FLWs) in India include Accredited Social Health Activists (ASHA), Anganwadi Workers (AWW) and Auxiliary Nurse Midwives (ANM), who are the three pillars within the community for the provision of basic health and nutrition services through demand generation and mobilization of beneficiaries to health services [25]. ASHA, a female health activist in the community caters for about 1000 population. Her major roles include creating health awareness, providing counselling to pregnant women, mobilizing women to community outreach services, accompaning pregnant women to health facility for delivery and providing postpartum care to mothers and newborns through home visits. The AWW provides health and nutrition education to the women and children and distributes supplementary nutrition whereas the ANM manages the sub-health centre (catering for a population about 5000) and provides services like immunization, antenatal care, postnatal care and basic management of sick children and primary healthcare services to the local population. ANM is also mandated to provide outreach services in her jurisdiction with support from AWW and ASHA. Detailed roles and responsibilities of each FLWs are elaborated elsewhere [25]. At the community level, the UPTSU supported AWWs and ASHAs (through mentoring and on-the-job support) to provide high-quality counselling on breastfeeding, including the importance of early initiation of breastfeeding, to pregnant women. Similarly, at the facility level, the UPTSU provided mentoring support to staff nurses to improve their skills and practices around birth including encouraging and providing breastfeeding support to mothers.

### Study design and participants

This study uses cross-sectional data from the Community Behaviour Tracking Survey (CBTS) implemented by the UPTSU and conducted between June–October 2018 in rural areas in the 25 HPDs of UP. The CBTS was designed to produce various indicators of Reproductive, Maternal, Newborn and Child Health (RMNCH) intervention at CD block, focused/non-focused CD block, and HPD level. Accordingly, four population subgroups were chosen for data collection to measure different indicators under RMNCH. Group-1 survey questionnaire was administered to the women with a pregnancy outcome of live birth, stillbirth or abortion in the 59 days preceding the survey, capturing antenatal care, childbirth, newborn care, postnatal care, and home based newborn care related information. This timeframe was chosen to obtain the most recent information and to minimize recall bias. Group-2 survey questionnaire was administered to the women with a child aged 60–179 days, to collect information on exclusive breastfeeding and child health. Group-3 survey questionnaire was administered to the women with a child aged 180–364 days, capturing information regarding initiation of complementary feeding practices. Group-4 survey questionnaire was administered to the women with a child aged 365–729 days to collect dietary diversity pattern under child health and nutrition.

The survey was conducted in 40 randomly selected CD blocks within the 25 HPDs. Of the 40 CD blocks, 20 CD blocks were selected randomly from the 100 focused CD blocks with the rest randomly selected from the remaining CD blocks (194) of 25 HPDs. Using a simple random sampling approach, 2811 primary sampling units (PSUs) within the selected CD blocks were chosen for one of the four survey groups – survey group with a maximum required number of PSUs. ASHA’s catchment area, which is the smallest health service delivery unit catering a population of about 1000 was taken as the PSU. The required number of PSUs for the remaining survey groups were randomely selected from the already selected 2811 PSUs. Within the PSU, all households were visited to identify eligible women for the respective survey groups and all the available eligible women were interviewed. A framework depicting this process is provided in Additional file 1. All four survey groups were canvased in focused CD blocks, while Group-1 and Group-2 survey groups were also canvased in non-focused CD blocks. For the present analysis, we used the data from Group-1 survey which had information related to early initiation of breastfeeding. In Group-1, a total of 13,908 women were identified as eligible and 12,041 were interviewed (86.6% response rate). Of these eligible women, 9124 aged 18 and above who had a live birth as pregnancy outcome in 59 days preceding the survey were considered for this analysis.

### Survey questionnaire

Group-1 survey questionnaire contained 172 questions to complete the interview in about 45 min. The survey questionnaire was divided into four broad sections covering information on woman’s socio-economic and household characteristics, antenatal care and birth preparedness having a battery of information on the utilization of antenatal services during pregnancy, advice or counselling received from FLWs during pregnancy on various health behaviours including IFA (iron and folic acid supplement) consumption, maternal nutrition, early initiation of breastfeeding, exclusive breastfeeding, family planning, emergency birth preparedness plan, and utilization of healthcare facilities for childbirth. In the other sections, information on postnatal and newborn care including the timing of breastfeeding initiation, postnatal support to initiate breastfeeding, prelacteal feeding practices, clean cord care, skin to skin contact, birthweight, home-based newborn care, treatment of diarrhoea and pneumonia were collected. The survey tool also gathered information on reproductive and family welfare. Female research investigators were recruited and trained to administer questionnaire in the local language (*Hindi*). Most of the questions related to health outcomes were captured in the CBTS using already available context-specific pre-validated questionnaires for the surveys like National Family Health Survey, District Level Household and Facility Surveys etc. As an additional step, the pretesting was done among a similar group of women in one of the HPD in UP to ensure the information flow, meaning of the questions, skipping pattern and internal consistency and was amended after piloting. Informed verbal consent was obtained from the respondents before administering the questionnaire. Handheld mobile devices with Open Data Kit (ODK) based (Android) application were used for data collection during the interviews. In addition, as part of quality assurance mechanism, female supervisors were appointed to monitor and supervise the field work, including back-check of interviews.

### Definitions and measurements

Early initiation of breastfeeding was measured as the percentage of infants, 0–59 days old, who initiated breastfeeding within 1 h after birth. The timing of initiation of breastfeeding was calculated based on responses to the question “*How long after the birth did you put the child on the breast for breastfeeding?*”. The responses were recorded in hours and we generated a binary variable of early initiation of breastfeeding (1 = Yes; 0 = No) if the initiation of breastfeeding was within an hour of birth. The analysis focused on two primary factors associated with early initiation of breastfeeding, received prenatal counselling and postnatal support immediately after birth. The respondents were asked, “*During pregnancy, did you receive any counselling/advice from ASHA/AWW/ANM on starting breastfeeding immediately after delivery*?”. To capture the postnatal breastfeeding support, the respondents were asked, “*Did you receive any support (to hold the child properly and attach the child’s mouth to the breast for proper breastfeeding) immediately after delivery?*”. This physical support could have been provided by a healthcare provider/birth attendant (Traditional Birth Attendant, ASHA or family member). Combining the prenatal breastfeeding counselling and postnatal support, an interaction variable was computed and categorized into four groups, i.e., mothers who received: i) both; ii) only prenatal counselling iii) only postnatal support, and iv) none.

Relevant confounders were identified from the existing literature [14, 18, 20, 22, 26–31] and included in the model to understand the association of prenatal breastfeeding counselling and postnatal support on early initiation of breastfeeding. These included: place of delivery (public health facility, private health facility, home); prelacteal feeding (given, not given); skin-to-skin contact given within 1 h after birth (yes, no); mode of delivery (vaginal, caesarean); number of visits for antenatal check-ups (ANC) during pregnancy (no check-up, 1, 2, 3, or 4 + check-ups); FLW contact during pregnancy (received ASHA/AWW contact at home, or attended village health nutrition day [VHND] or visited a sub-centre to receive antenatal check-up (yes, no)); maternal age (< 20, 20–24, 25–29, 30+); years of schooling (non-literate, < 5 years, 5–10 years, 10 + years); parity (1, 2, 3, 4 +); caste (Scheduled Caste (SC), Scheduled Tribe (ST), Other Backward Class (OBC), Others); religion (Hindu, Non-Hindu); standard of living index (low, medium, high); and sex of the child (boy, girl). Prelacteal feeding refers to “giving the newborn anything (such as honey, water, tea, jaggery, local herbs or ‘*ghutti’*) before breast milk for the first time”. Skin-to-skin contact refers to “newborn laid directly on their mother’s bare chest after birth, and both of them covered in a cloth”.

Standard of living index was computed based on a composite score derived from a set of household indicators such as access to drinking water (tap water, hand pump/protected well, others), access to toilet facility (flush, pit, no toilet), type of dwelling (*pucca, semi-pucca, kaccha*) and ownership of household assets (electricity, black & white television, colour television, mobile, land telephone, refrigerator, air conditioner, bicycle, motorcycle, car, water pump, tractor) using principal component analysis. The composite score was divided into three equal categories, each with 33% households [32].

### Statistical analysis

**Fig. 1:**
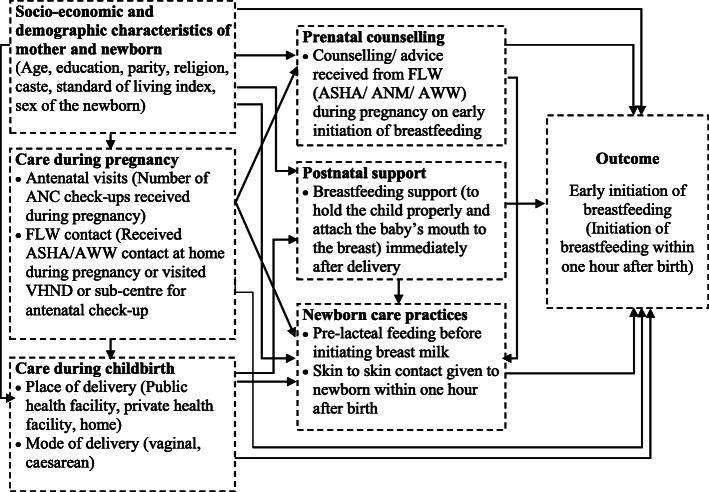
Analytical framework of early initiation of breastfeeding

We developed an analytical framework to assess the association of prenatal counselling regarding breastfeeding and postnatal support on early initiation of breastfeeding for this analysis (Fig. 1). Descriptive analysis was done to describe the background socio-demographic characteristics of the mothers. Bivariate analysis and multivariate logistic regressions were used to examine the association of prenatal breastfeeding counselling and postnatal support on early initiation of brestfeeding. Four separate models were built. While the first model was an overall model adjusting for the place of delivery, the remaining three models were separately built for each type of delivery point. Multivariate results are presented in the form of unadjusted and adjusted odds ratios (aOR), and 95% confidence interval (95% CI). Appropriate sampling weights were used. All analyses were conducted using STATA version 15.0 [33].

## Results

The median age of the 9124 mothers was 24 years and they had 2.6 children on average (Table 1). About 53.2% of the mothers were non-literate, 81.9% were Hindus, 26.9% belonged to SC category and 52.5% of the mothers had male children. Further, 31.8% received four or more antenatal appointments, 80.6% reported having had contact with a FLW during the prenatal period, and 7.0% of the mothers had a caesarean section. Overall, 73.5% of newborns were not given any prelacteal and 12.4% were given skin-to-skin contact. Results show that 59.9% had delivered in a public health facility, 17.9% in a private health facility and 22.2% at home. A higher percentage of mothers who delivered at home were non-literate (70.9%), had a higher number of children (3.3%), were non-Hindus (23.4%), and had a low standard of living index (42.0%) compared to those who delivered in a public or private health facility. Moreover, ANC, newborn and childbirth practices also differed significantly among mothers who delivered at public health facilities, private health facilities and home (Table 1).

**Table 1:** Profile of mothers stratified by place of delivery

		Place of delivery		
Characteristics	Overall	Public	Private	Home
**Socio-economic and demographic characteristics of mother**				
Median age (years) [IQR]	24 [5]	24 [6]	24 [5]	25 [7]
Mean number of children	2.6	2.5	2.2	3.3
% non-literate	53.2	51.5	37.1	70.9
% belonged to Hindu religion	81.9	85.1	78.0	76.6
% belonged to SC category	26.9	29.9	19.0	25.0
% belonged to OBC category	57.7	56.1	58.3	61.6
% belonged to low standard of living	32.1	32.7	17.7	42.0
% belonged to middle standard of living	34.5	35.6	29.3	35.6
% had a boy child	52.5	52.9	52.4	51.5
**Care during pregnancy**				
% received 4 or more ANC check-ups	31.8	33.0	49.7	14.1
% had any FLW contact	80.6	85.6	75.8	71.2
**Care during childbirth**				
Place of delivery	–	59.9	17.9	22.2
% caesarean delivery	7.0	1.2	34.5	0.0
**Newborn care**				
% not given any pre-lacteal	73.5	88.3	50.1	52.3
% received skin-to-skin contact within an hour after birth	12.4	18.1	4.5	3.2
**Prenatal counselling and postnatal support**				
% received *both* prenatal counselling and post-natal support	27.2	33.0	22.1	15.6
% received *only* prenatal counselling	12.7	12.3	11.5	14.6
% received *only* post-natal support	31.9	31.6	38.4	27.5
% received *none*	28.2	23.1	28.0	42.2
***N***	**9124**	**5302**	**1617**	**2205**

Note: IQR refers interquartile range

Overall, 27.2% of mothers received both prenatal counselling and postnatal support, 12.7% received only prenatal counselling, 31.9% received only postnatal support and 28.2% received none. Exposure to the interventions differed by place of delivery. For example, a higher proportion of mothers who received both interventions delivered at a public health facility (33.0%), followed by private (22.1%) and home (15.6%). In contrast, a higher percentage of mothers who delivered at home received none of the interventions (42.2%) compared to mothers who delivered at public (23.1%) or private health facilities (28.0%).

### Association of prenatal counselling and postnatal support with early initiation of breastfeeding

**Fig. 2:**
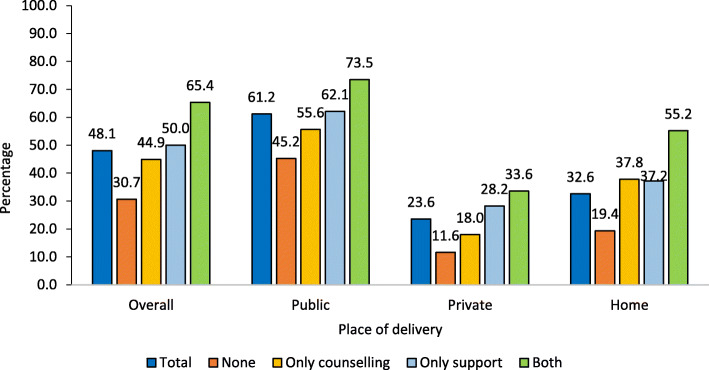
Percentage of mothers who reported early initiation of breastfeeding following prenatal counselling and postnatal support, stratified by place of delivery

**Fig. 3:**
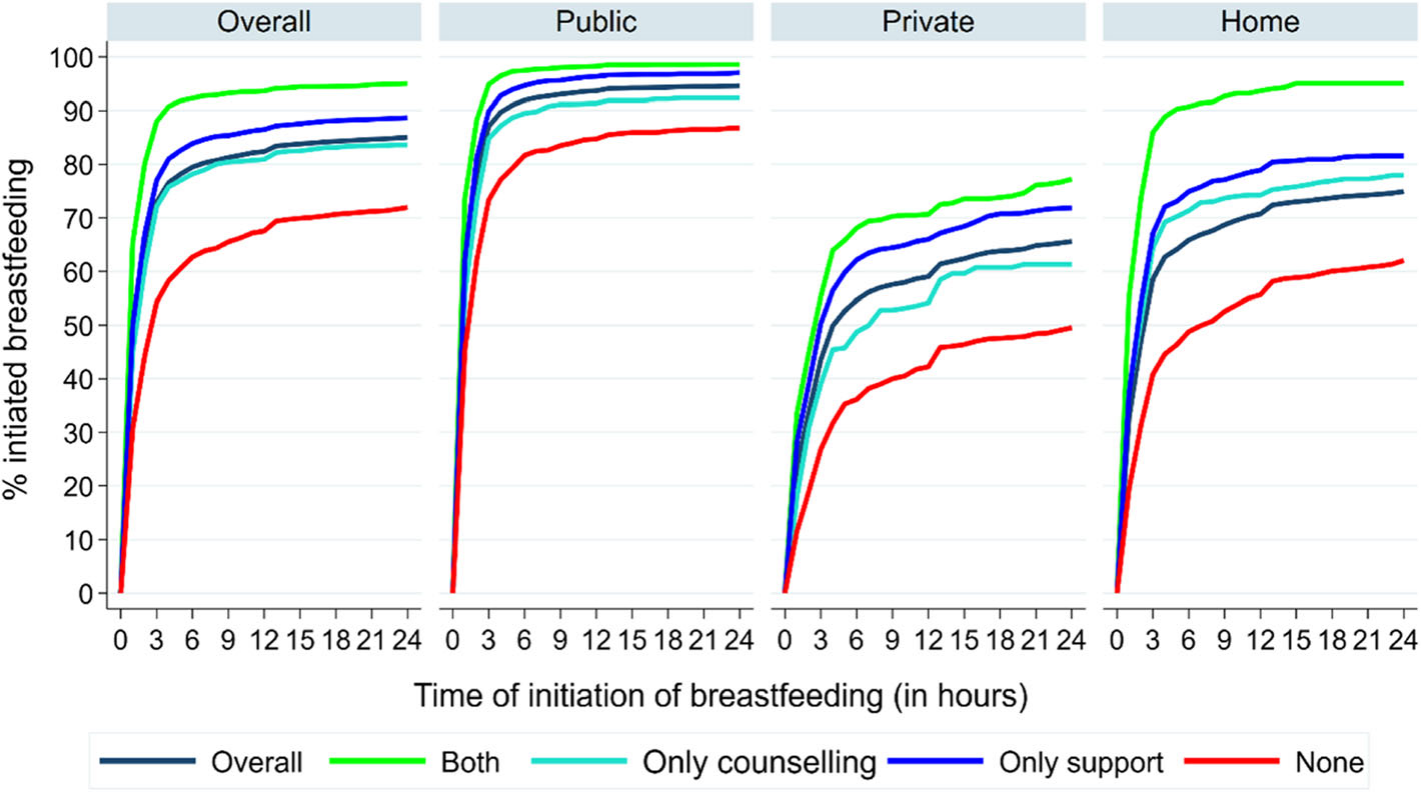
Cumulative distribution of percentage of mothers who initiated breastfeeding by time and intervention (prenatal counselling and postnatal support), stratified by place of delivery

Nearly half of the mothers (48.1%) reported initiating breastfeeding within 1 h of birth (Fig. 2). Early initiation of breastfeeding was highest among those who received both prenatal counselling and postnatal support (65.4%), and lowest among mothers who received none (30.7%). Early initiation of breastfeeding was 44.9% among those who received only prenatal counselling and 50.0% among those who received only postnatal support. Early initiation of breastfeeding rates were 61.2, 23.6 and 32.6% among those delivered in a public health facility, private health facility, and home respectively. The results further show that, for each of the mentioned places of delivery, the early initiation of breastfeeding remained highest among those who received both interventions (73.5, 33.6, 55.2%) followed by those who received only postnatal support, and only prenatal counselling compared to none. Figure 3 also reveals that even if we consider those who initiated breastfeeding within 24 h, instead of within an hour, breastfeeding initiation was highest among those mothers who received both the interventions followed by those received either postnatal support, or prenatal counselling compared to none in all the three places of delivery. Further, the largest positive effect of the interventions on initiating breastfeeding within 24 h was observed among home deliveries, followed by deliveries at private health facilities. Differentials in percentage newborn received early initiation of breastfeeding by socio-economic and demographic characteristics of the mother, care during pregnancy, care during childbirth and newborn care are presented in appendix (Additional file 2).

The results of logistic regression analyses in Table 2 show that the unadjusted odds ratio (OR) corroborated the bivariate findings. The highest likelihood of early initiation of breastfeeding was found among mothers who received both the interventions (OR 4.26; 95% CI 3.75, 4.85) compared to none. Similarly, the odds of early initiation of breastfeeding was 2.26 (95% CI 2.01, 2.55) and 1.84 (95% CI 1.58, 2.15) among those who received only postnatal support and only prenatal counselling, respectively, compared to none.

**Table 2:** Un-adjusted and adjusted odds ratios (95% CI) of early initiation of breastfeeding by counselling and support, stratified by place of delivery

	Un-adjusted Odds Ratios [95% CI] by place of delivery				Adjusted Odds Ratios [95% CI] by place of delivery			
	Overall	Public	Private	Home	Model I (Overall)	Model II (Public)	Model III (Private)	Model IV (Home)
**Place of delivery**								
Home [Ref]								
Public	3.26*** (2.91, 3.66)				1.53*** (1.33, 1.75)			
Private	0.64*** (0.55, 0.75)				0.69*** (0.56, 0.83)			
**Prenatal counselling**								
No [Ref]								
Yes	2.06*** (1.88, 2.27)	1.79*** (1.59, 2.03)	1.46*** (1.13, 1.90)	2.45*** (1.99, 3.01)	1.37*** (1.22, 1.53)	1.34*** (1.16, 1.54)	1.22 (0.87, 1.70)	1.63*** (1.27, 2.10)
**Postnatal support**								
No [Ref]								
Yes	2.46*** (2.24, 2.70)	2.22*** (1.96, 2.51)	2.79*** (2.10, 3.69)	2.45*** (2.01, 2.98)	1.96*** (1.75, 2.18)	1.87*** (1.63, 2.15)	2.83*** (2.05, 3.92)	1.83*** (1.46, 2.30)
**Interaction of prenatal counselling and postnatal support**								
None [Ref]								
Only counselling	1.84*** (1.58, 2.15)	1.52*** (1.24, 1.86)	1.67*** (1.01, 2.76)	2.53*** (1.88, 3.41)	1.40*** (1.18, 1.67)	1.24** (1.00, 1.54)	1.36 (0.76, 2.43)	2.00*** (1.41, 2.78)
Only support	2.26*** (2.01, 2.55)	1.98*** (1.69, 2.33)	3.00*** (2.11, 4.27)	2.47*** (1.92, 3.17)	1.99*** (1.73, 2.28)	1.79*** (1.50, 2.13)	3.00*** (2.01, 4.47)	2.11*** (1.59, 2.79)
Both	4.26*** (3.75, 4.85)	3.36*** (2.84, 3.97)	3.86*** (2.65, 5.63)	5.13*** (3.84, 6.85)	2.67*** (2.30, 3.11)	2.49*** (2.07, 3.01)	3.50*** (2.25, 5.44)	2.84*** (2.02, 3.98)

Note: Models are adjusted for prelacteal feeding, caesarean delivery, skin-to-skin contact within 1 hour of delivery, number of ANC check-ups, FLW contact during pregnancy, age, education, parity, religion, caste, standard of living index, sex of the child and place of delivery. Models 2–4 are not adjusted for place of delivery

*CI* Confidence interval, *Ref* Reference Category

***p < 0.01, **p < 0.05

Multivariate results show that after adjusting for variables such as the socio-economic and demographic characteristics of the mother, ANC check-ups, FLW contact, place of delivery, type of delivery, and newborn care practices, the aOR of early initiation of breastfeeding was 1.37 (95% CI 1.22, 1.53) among mothers who had received prenatal counselling and 1.96 (95% CI 1.75, 2.18) among those received postnatal support compared to their respective counterparts. After introducing an interaction between prenatal counselling and postnatal support in the model, the aOR of early initiation of breastfeeding was 2.67 (95% CI 2.30, 3.11) if the mother received both interventions, 1.40 (95% CI 1.18, 1.67) if the mother received only prenatal counselling, and 1.99 (95% CI 1.73, 2.28) if the mother received only postnatal support compared to the mothers who received none.

Compared to mothers who did not receive prenatal counselling, the aOR of early initiation of breastfeeding among mothers who received prenatal counselling was 1.34 (95% CI 1.16, 1.54) among mothers delivered in public health facilities, and 1.63 (95% CI 1.27, 2.10) among mothers delivered at home after adjusting for the a priori variables identified above. Similarly, compared to mothers who did not receive postnatal support, the likelihood of early initiation of breastfeeding among mothers who received postnatal support ranged from 1.87 (95% CI 1.63, 2.15) among mothers delivered in public health facilities, 2.83 (95% CI 2.05, 3.92) among mothers delivered in private facilities and 1.83 (95% CI 1.46, 2.30) among mothers who delivered at home.

The aOR of early initiation of breastfeeding in Models 11, 111, 1 V including the interaction of both the interventions depicted that the likelihood of early initiation of breastfeeding among mothers who delivered at public health facilities was 2.49 (95% CI 2.07, 3.01) if mothers received both interventions, 1.24 (95% CI 1.00, 1.54) if mothers received only prenatal counselling, and 1.79 (95% CI 1.50, 2.13) if mothers received only postnatal support compared to those who received none. Among mothers who delivered at private health facilities, the aOR of early initiation of breastfeeding was 3.50 (95% CI 2.25, 5.44) if mothers received both, and 3.00 (95% CI 2.01, 4.47) if mothers received only postnatal support compared to none. Among mothers who delivered at home, the aOR of early initiation of breastfeeding was 2.84 (95% CI 2.02, 3.98) if mothers received both, 2.00 (95% CI 1.41, 2.78) if mothers received only prenatal counselling, and 2.11 (95% CI 1.59, 2.79) if mothers received only postnatal support compared to none.

## Discussion

Overall, less than half of mothers (48%) reported initiation of breastfeeding within 1 h of delivery. The study identified a significant association of two important interventions of receiving prenatal counselling on breastfeeding and postnatal support to mothers, with early initiation of breastfeeding. Mothers who received both counselling and support were 2.7 times more likely to initiate early breastfeeding. In addition, irrespective of the place of delivery, the likelihood of early initiation of breastfeeding was significantly higher among mothers who received both the interventions, followed by either one of the interventions compared to those who were not exposed to any of the interventions.

Similar to other studies that have demonstrated the counselling by a dedicated, trained breastfeeding counsellor during pregnancy is associated with higher rates of early initiation of breastfeeding [12, 34], we also found that providing breastfeeding counselling by FLWs anytime during pregnancy had a positive association with early initiation of breastfeeding. Moreover, prenatal counselling on breastfeeding (as a separate intervention) showed significant association with early initiation of breastfeeding among mothers who delivered at public health facilities, but not among mothers who delivered at private health facilities. This could be due to a greater possibility of contacts/home visits by FLWs leading to more counselling opportunities among mothers who delivered at public facilities compared to private facilities. Women belonging to a higher standard of living strata are more likely to deliver in private facilities, less likely to have contact with FLWs and may also have a bias towards non-breastfeeding based on cultural exposure or practice [35]. Hence, it is important to ensure that breastfeeding counselling during pregnancy is provided to all pregnant women irrespective of their socio-economic status.

The study also showed that mothers receiving postnatal support (as a separate intervention) immediately after birth had a significantly higher likelihood of initiating breastfeeding within an hour. Even after adjusting for the place of delivery and other covariates, the likelihood of early initiation of breastfeeding increased by almost two-fold when mothers received postnatal support at the place of birth. Very few studies have analysed the effect of immediate postnatal support on the initiation of breastfeeding [36]. Postnatal support on breastfeeding to mothers by healthcare providers immediately after delivery helps mothers to initiate and establish breastfeeding, as well as manage common breastfeeding difficulties during this critical period [37]. A higher likelihood of early initiation of breastfeeding among mothers receiving postnatal support over prenatal counselling, especially in public health facilities, may also be attributed to better implementation of the Infant Milk Substitutes (IMS), Feeding Bottles and Infant Foods a regulation of production, supply and distribution Act (IMS Act) that aims to protect and promote breastfeeding in public health facilities compared to private health facilities [38]. Interestingly, our study found that 43.3% of mothers who delivered at home reported receipt of postnatal support. We found that nearly half of the home deliveries (data not shown) were attended by a *Dai,* a traditional birth attendant, and the remainder were attended by an older family member. Evidence suggests that traditional birth attendants play a crucial role in providing care around birth including promoting breastfeeding practices in rural areas in India [34, 39].

In the overall model, the likelihood of early initiation of breastfeeding was almost three-times higher among mothers who received both the interventions compared to none. Also between the two interventions – counselling and support – the magnitude of association of the latter with early initiation of breastfeeding was higher compared to prenatal counselling (aOR 1.99 versus 1.40). This suggests that while prenatal counselling is important, providing breastfeeding support to mothers immediately after birth could have a greater positive association with early initiation of breastfeeding. The early initiation of breastfeeding was substantially higher in public health facilities compared to private health facilities. This could be explained by the higher levels of caesarean sections and prelacteal feeding practices in private health facilities as compared to public health facilities in India which have been previously demonstrated to be associated with lower early initiation of breastfeeding [14, 17, 19, 22]. In UP, access to private health facilities for childbirth increased substantially from 14% in 2005–06 to 23% in 2015–16 and the main contribution of caesarean section delivery in the state is from the private sector [10]. However, even after adjusting for caesarean section and prelacteal feeding along with other covariates, the likelihood of early initiation of breastfeeding among mothers who delivered in private health facilities remained lower (by 30%) even compared to mothers who delivered at home.

This study has a few important limitations. The study findings are based on the data from only 25 out of 75 districts in UP. Additionally, the cross-sectional nature of the study precludes any causal interpretations between the interventions and early initiation of breastfeeding. The study anticipated low recall bias by using a short reference period (59 days preceding survey) to capture recent deliveries. However, recall bias cannot be definitively ruled out and this could have affected the estimated prevalence of early initiation of breastfeeding. The cultural confounders like the belief about lack of breast milk, practice of initiation of colostrum etc. that are critical to effecting behaviour change of the mother and healthcare provider’s were not captured in the survey and hence not included in the analysis. The facility-level information on labour room practices that promote or inhibit early initiation of breastfeeding, including skill and knowledge of the healthcare provider on breastfeeding postnatal support, were not available. The quality of antenatal counselling by FLWs and postnatal support on breastfeeding immediately after delivery was not examined in this study. The study also could not consider the role of preterm babies on early initiation of breastfeeding rates.

Despite these limitations, the results from the study are generalizable for the entire state of UP considering the nature of the intervention. Further research is required to understand the role of quantity, quality, and timing of prenatal counselling and postnatal support in enhancing the levels of early initiation of breastfeeding. In addition, research is also required to understand why early initiation of breastfeeding rates are so low in private health facilities of 25 HPDs in UP apart from higher levels of caesarean section and prelacteals as observed in this study, given the increase in proportion of deliveries in private sector in recent years.

## Conclusion

This study finds that 52% of mothers do not initiate breastfeeding wihin an hour after delivery and that providing both prenatal counselling on early initiation of breastfeeding and postnatal support immediately after birth together may reduce this gap substantially. Given that only 27% of mothers received both the interventions, programmatic efforts are needed to saturate the coverage of these two interventions through community and facility-based platforms.

As an intervention, community-based service delivery points for ANC, such as Village Health Nutrition Days (VHNDs), home visits made by ASHA/AWW and facilities providing ANC should be utilized to ensure that all pregnant women receive counselling on the importance of timely initiation of breastfeeding and its numerous benefits. In addition, health facilities and home deliveries need to provide postnatal support to all mothers immediately after birth to better promote early initiation of breastfeeding. Adequate training of healthcare providers and traditional birth attendants on postnatal breastfeeding support including position, attachment and managing breastfeeding complications could be effective strategies in increasing early initiation of breastfeeding. Lastly, the strict implementation of the IMS Act in private health facilities will promote early initiation of breastfeeding. The impact of a conducive policy environment on protecting, promoting and supporting breastfeeding cannot be overstated. Targeted policy advocacy and decisions are needed to improve early initiation of breastfeeding, thereby reducing neonatal mortality and achieving the Sustainable Development Goals.

## Supplementary Information

**Additional file 1:.** Framework of sample selection.

**Additional file 2:.** Percentage of newborns who received early initiation of breastfeeding by background characteristics.

**Additional file 3:.** Characteristics of mothers according to classification of receiving prenatal counselling and postnatal support.

**Additional file 4:.** Nutrition and health counselling booklet.

## Data Availability

The datasets used and/or analysed during the current study are available from the corresponding author on reasonable request.

## Abbreviations

ANC: Antenatal Check-ups
aOR: Adjusted Odd Ratio
ANM: Auxiliary Nurse Midwifery
ASHA: Accredited Social Health Activist
AWW: Anganwadi Worker
CBTS: Community Behaviour Tracking Survey
CD: Community Development
CI: Confidence Interval
FLW: Frontline Worker
GOI: Government of India
GOUP: Government of Uttar Pradesh
HPD: High Priority Districts
IMS: Infant Milk Substitutes
IQR: Inter Quartile Range
NMR: Neonatal Mortality Rate
OBC: Other Backward Class
ODK: Open Data Kit
PSU: Primary Sampling Unit
SC: Scheduled Caste
ST: Scheduled Tribe
UNICEF: United Nations Children Fund
UP: Uttar Pradesh
UPTSU: Uttar Pradesh Technical Support Unit
VHND: Village Health Nutrition Day
WHO: World Health Organization
