# Supplementary material for: Association of prenatal counselling and immediate postnatal support with early initiation of breastfeeding in Uttar Pradesh, India
Source: Int Breastfeed J. 2021 Mar 16;16:26. doi: 10.1186/s13006-021-00372-6 (PMC7968284; doi:10.1186/s13006-021-00372-6)
Supplement: Supplementary file 1 — Additional file 1:. Framework of sample selection. [file 13006_2021_372_MOESM1_ESM.docx]

25 High Priority Districts, UP

(294 CD Blocks)

100 focused CD Blocks

194 non-focused CD Blocks

20 CD Blocks selected randomly

20 CD Blocks selected randomly

All available eligible women (of different groups) in the PSU were interviewed.

(12041 in group-1, 13534 in group-2, 6826 in group-3 and 6966 in group-4 were interviewed)

In each PSU all the households were visited to identify eligible women for respective survey groups.

Group-1: women with pregnancy outcome of live birth, stillbirth or abortion in 0-59 days

Group-2: women with child aged 60-179 days

Group-3: women with child aged 180-364 days

Group-4: women with child aged 365-729 days

Present analysis was carried out among 9124 eligible women who had a live birth in 0-59 days preceding survey from group-1 data.

1362 PSUs (ASHA catchment areas) were selected randomly for one of four survey groups with maximum required number of PSUs from a complete listing of ASHA areas in the CD block

1449 PSUs (ASHA catchment areas) were selected randomly for one of four survey groups with maximum required number of PSUs from a complete listing of ASHA areas in the CD block

PSUs for the remaining survey groups were randomly selected from the already selected PSUs.

Out of 1449 selected PSUs, 1334 PSUs for group-1 and 1166 PSUs for group-2 were selected randomly

PSUs for the remaining survey groups were randomly selected from the already selected PSUs.

Out of 1362 selected PSUs, 1312 PSUs for group-1, 1156 PSUs for group-2, 617 PSUs for group-3, and 428 PSUs for group-4 were selected.
