# Supplementary material for: Association of prenatal counselling and immediate postnatal support with early initiation of breastfeeding in Uttar Pradesh, India
Source: Int Breastfeed J. 2021 Mar 16;16:26. doi: 10.1186/s13006-021-00372-6 (PMC7968284; doi:10.1186/s13006-021-00372-6)
Supplement: Supplementary file 2 — Additional file 2:. Percentage of newborns who received early initiation of breastfeeding by background characteristics. [file 13006_2021_372_MOESM2_ESM.docx]

| **Characteristics** |  | **Place of delivery** | | |
| --- | --- | --- | --- | --- |
|  | **Overall** | **Public** | **Private** | **Home** |
| **Socio-economic and demographic characteristics of mother** |  |  |  |  |
| **Age of women** |  |  |  |  |
| *<20* | 46.8 | 57.2 | 22.5 | 31.3 |
| *20-24* | 49.4 | 62.1 | 22.7 | 32.6 |
| *25-29* | 48.7 | 62.1 | 26.6 | 33.3 |
| *30+* | 43.5 | 57.8 | 19.8 | 31.6 |
| *Chi-square (p value)* | *0.005* | *0.155* | *0.236* | *0.654* |
| **Parity** |  |  |  |  |
| *1* | 48.3 | 59.9 | 23.2 | 33.8 |
| *2* | 50.1 | 64.1 | 24.0 | 33.4 |
| *3* | 48.7 | 62.4 | 21.9 | 33.6 |
| *4+* | 45.2 | 58.6 | 25.4 | 30.9 |
| *Chi-square (p value)* | *0.024* | *0.012* | *0.705* | *0.554* |
| **Years of schooling** |  |  |  |  |
| *No education / <5 years* | 46.6 | 59.4 | 22.4 | 31.7 |
| *5-10 years* | 49.6 | 61.9 | 23.5 | 34.7 |
| *10+ years* | 50.2 | 65.0 | 25.0 | 35.1 |
| *Chi-square (p value)* | *0.020* | *0.009* | *0.473* | *0.399* |
| **Religion** |  |  |  |  |
| *Hindu* | 49.5 | 61.3 | 24.6 | 34.7 |
| *Non-Hindu* | 41.5 | 60.4 | 19.9 | 25.7 |
| *Chi-square (p value)* | *0.000* | *0.513* | *0.202* | *0.006* |
| **Caste** |  |  |  |  |
| *SC/ST* | 51.7 | 61.6 | 27.1 | 35.1 |
| *OBC* | 47.8 | 61.8 | 24.4 | 31.1 |
| *Others* | 41.7 | 57.1 | 18.2 | 34.4 |
| *Chi-square (p value)* | *0.000* | *0.097* | *0.074* | *0.225* |
| **Standard of living** |  |  |  |  |
| *Poor* | 48.2 | 60.1 | 25.2 | 30.9 |
| *Middle* | 49.4 | 61.1 | 25.3 | 34.1 |
| *Rich* | 46.6 | 62.4 | 22.1 | 33.3 |
| *Chi-square (p value)* | *0.084* | *0.411* | *0.376* | *0.292* |
| **Sex of the child** |  |  |  |  |
| *Boy* | 49.2 | 62.2 | 24.2 | 33.8 |
| *Girl* | 46.8 | 60.0 | 22.9 | 31.3 |
| *Chi-square (p value)* | *0.013* | *0.037* | *0.683* | *0.260* |
| **Availing care during pregnancy** |  |  |  |  |
| Received four or more ANC |  |  |  |  |
| *No* | 46.9 | 59.7 | 23.3 | 31.0 |
| *Yes* | 50.7 | 64.2 | 23.8 | 42.4 |
| *Chi-square (p value)* | 0.006 | 0.003 | 0.464 | 0.000 |
| Received FLW contact |  |  |  |  |
| *No* | 33.5 | 47.7 | 22.6 | 21.8 |
| *Yes* | 51.6 | 63.5 | 23.9 | 36.9 |
| *Chi-square (p value)* | *0.000* | *0.000* | *0.860* | *0.000* |
| **Availing care during childbirth** |  |  |  |  |
| Mode of delivery |  |  |  |  |
| *Normal* | 50.9 | 61.5 | 31.7 | 32.6 |
| *Caesarean* | 11.3 | 38.0 | 8.2 | 11.7 |
| *Chi-square (p value)* | *0.000* | *0.000* | *0.000* | *0.000* |
| **Newborn care** |  |  |  |  |
| Received any pre-lacteal |  |  |  |  |
| *Yes* | 12.1 | 21.8 | 5.5 | 11.3 |
| *No* | 61.1 | 66.4 | 41.5 | 52.0 |
| *Chi-square (p value)* | *0.000* | *0.000* | *0.000* | *0.000* |
| Received skin-to-skin contact within an hour after birth |  |  |  |  |
| *No* | 44.5 | 58.2 | 22.0 | 31.4 |
| *Yes* | 73.2 | 74.6 | 58.2 | 68.3 |
| *Chi-square (p value)* | *0.000* | *0.000* | *0.000* | *0.000* |
| **Prenatal counselling and postnatal support** |  |  |  |  |
| Received prenatal counselling |  |  |  |  |
| *No* | 41.0 | 55.0 | 21.2 | 26.4 |
| *Yes* | 58.9 | 68.7 | 28.2 | 46.8 |
| *Chi-square (p value)* | *0.000* | *0.000* | *0.001* | *0.000* |
| Received postnatal support |  |  |  |  |
| *No* | 35.1 | 48.9 | 13.4 | 24.1 |
| *Yes* | 57.1 | 67.9 | 30.2 | 43.7 |
| *Chi-square (p value)* | *0.000* | *0.000* | *0.000* | *0.000* |
