# Supplementary material for: Association of prenatal counselling and immediate postnatal support with early initiation of breastfeeding in Uttar Pradesh, India
Source: Int Breastfeed J. 2021 Mar 16;16:26. doi: 10.1186/s13006-021-00372-6 (PMC7968284; doi:10.1186/s13006-021-00372-6)
Supplement: Supplementary file 3 — Additional file 3:. Characteristics of mothers according to classification of receiving prenatal counselling and postnatal support. [file 13006_2021_372_MOESM3_ESM.docx]

| **Characteristics** | **Received both prenatal counselling and postnatal support (N=2375)** | **Received only prenatal counselling (N=1187)** | **Received only postnatal support (2799)** | **Received neither counselling nor postnatal support (28.2%; N=2763)** |
| --- | --- | --- | --- | --- |
| **Socio-demographic characteristics** |  |  |  |  |
| Age, years |  |  |  |  |
| *% aged <20 years* | 5.8 | 4.1 | 7.9 | 5.2 |
| *% aged 20-29 years* | 81.8 | 78.5 | 78.2 | 74.3 |
| *% aged 30 years and above* | 12.3 | 17.4 | 14.0 | 20.5 |
| % with 4+ birth order | 19.0 | 28.6 | 20.3 | 31.5 |
| % non-literate | 47.9 | 58.2 | 51.0 | 62.4 |
| % belonged to low standard of living | 27.7 | 31.1 | 31.6 | 37.4 |
| % belonged to same caste group of ASHA | 50.0 | 52.9 | 44.0 | 48.6 |
| **Care during pregnancy and childbirth** |  |  |  |  |
| % received any ANC | 95.4 | 92.4 | 87.9 | 80.3 |
| % received 4 or more ANC check-ups | 42.8 | 31.8 | 32.2 | 20.6 |
| % delivered at home | 12.8 | 25.7 | 19.1 | 33.2 |
