# Supplementary material for: Association of prenatal counselling and immediate postnatal support with early initiation of breastfeeding in Uttar Pradesh, India
Source: Int Breastfeed J. 2021 Mar 16;16:26. doi: 10.1186/s13006-021-00372-6 (PMC7968284; doi:10.1186/s13006-021-00372-6)
Supplement: Supplementary file 4 — Additional file 4:. Nutrition and health counselling booklet. [file 13006_2021_372_MOESM4_ESM.pdf]

स्वच्छ भारत
एक कदम स्वच्छता की ओर

उत्तर प्रदेश सरकार

राज्य पोषण मिशन

NATIONAL HEALTH MISSION
राष्ट्रीय स्वास्थ्य मिशन

परामर्श पुस्तिका

(पोषण व स्वास्थ्य सम्बंधित परामर्श संदेश)

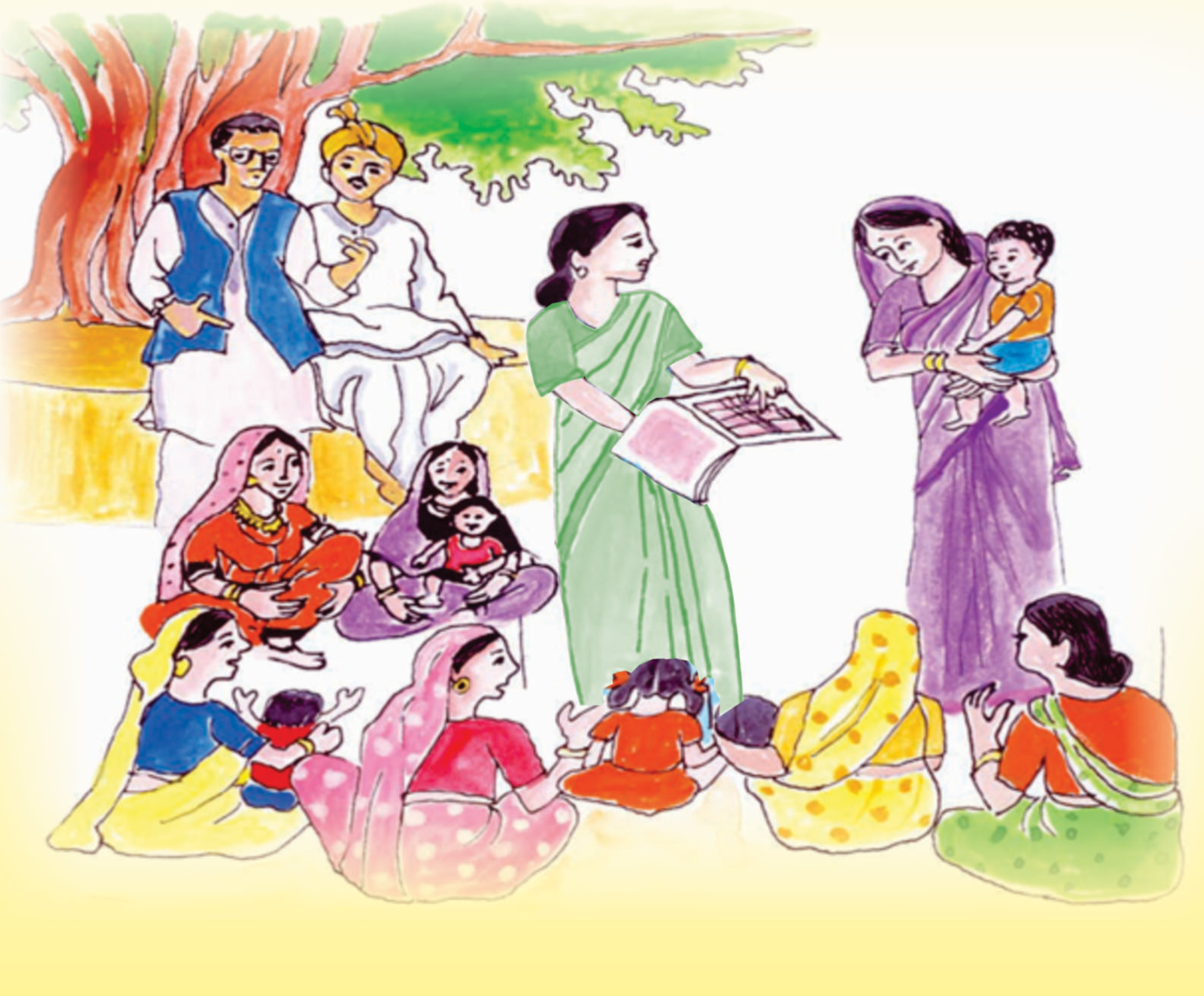

इस पुस्तिका का प्रयोग

☛ **यह पुस्तिका मातृ एवं शिशु स्वास्थ्य व पोषण संबंधी संदेशों को देने एवं इसके माध्यम से व्यवहारों को अपनाने व सुधार लाने हेतु बनायी गयी है।**

☛ **समूह चर्चा एवं गृह भ्रमण के दौरान माताओं को सही जानकारी देने के लिए इस पुस्तिका को ध्यान से पढ़ें एवं उपयोग करें।**

★ ये पुस्तिका किसके द्वारा प्रयोग की जानी है?

इस पुस्तिका का प्रयोग निम्न अग्रिम पंक्ति के कार्यकर्ताओं द्वारा किया जा सकता है।

- आंगनवाड़ी कार्यकत्री
- आशा
- ए.एन.एम.

★ परामर्श किस–किस को दिया जा सकता है?

- गर्भवती /धात्री महिलाओं को।
- दो वर्ष तक के बच्चों तथा गम्भीर अल्प वजन (लाल श्रेणी) वाले पांच वर्ष तक के बच्चों की माताओं को।
- घर के अन्य सदस्य–सास, पति को ।
- ग्राम प्रधान एवं मातृ समिति के सदस्यों को।

★ परामर्श किन–किन मुद्दों पर दिया जा सकता है?

1. गर्भवती एवं धात्री माताओं का पोषण।
2. 0–6 माह तक के बच्चों में शीघ्र व केवल स्तनपान।
3. 6 माह से 2 साल तक के बच्चों में पूरक आहार
4. बच्चो में सूक्ष्म पोषक तत्वों का सम्पूरण।
5. बच्चो में वृद्धि निगरानी।
6. स्वच्छता एवं साफ़–सफाई।
7. प्रजनन स्वास्थ्य।

★ परामर्श कब–कब दिया जा सकता है?

- महिने में कम से कम सात दिन–
  तीन दिन आंगनवाड़ी केंद्र पर पूरक आहार के वितरण के दौरान हर महीने की 5, 15 एवं 25 तारीख को या फिर आई.सी.डी.एस. द्वारा निधारित दिवस पर।
- चार सप्ताहिक दिवस, सप्ताह में एक–एक बार किसी भी दिन, जब भी गर्भवती माहिला और बच्चे माँ के साथ आंगनवाड़ी केंद्र पर खाना खाने के लिये आएंगे।
- ग्राम स्वास्थ्य पोषण दिवस के अवसर पर।
- गृह भ्रमण के दौरान।

★ परामर्श देने का तरीका क्या होना चाहिए?

– परामर्श देते समय निम्न बातों का ध्यान रखना चाहिएः–

- पहले अभिवादन करना।
- फिर प्रश्न पूछना।
- उनको बताना।
- उनकी सहायता करना।
- उनकों समझाना।
- दोबारा मिलकर पहले दिये संदेशों को दोहराना।

- **समूह चर्चा के दौरान परामर्श एवं प्रदर्शन**– समूह चर्चा के दौरान सभी लाभार्थियों को एक घेरे में बिठाये, परामर्श दाता संवाद शुरू करने के लिए परामर्श विषय संबंधित कुछ प्रश्न पूछें और समूह में उपस्थित लोग अपने अनुभवों को सब के साथ बाटें, इसके आधार पर ही परामर्शदाता सही व्यवहारों और संदेशों को समूह के लोगों को समझाने में सहायता करें।

- **गृह भ्रमण के दौरान परामर्श एवं प्रदर्शन**–गृह भ्रमण के दौरान समूह चर्चा में दिये गये परामर्श को दोहराएं और हर लाभार्थी की स्वास्थ्य एवं पोषण संबंधी समस्याओं का भी समाधान करने में उनकी सहायता करें।

अध्याय–1

गर्भवती एवं धात्री महिला का पोषण

**गर्भावस्था में उचित पोषण से महिला स्वस्थ्य रहती है,
एक स्वस्थ्य महिला ही एक स्वस्थ्य शिशु को जन्म देती है।**

✦ **समूह चर्चा के दौरान संवाद शुरू करने के लिए पूछे जाने वाले प्रश्न तथा उनसे सम्बंधित संदेशः**

❊ **गर्भवती एवं धात्री महिला को पोषण के बारे में किन–किन बातों का ध्यान रखना चाहिये?**

★ **संदेशः**

– **गर्भवती एवं धात्री महिलाओं के आहार की विविधता व मात्रा**

- नीचे दी गई तालिका में से कम से कम चार या उससे अधिक खाद्य समूहों को दैनिक भोजन में शामिल करने को कहें।

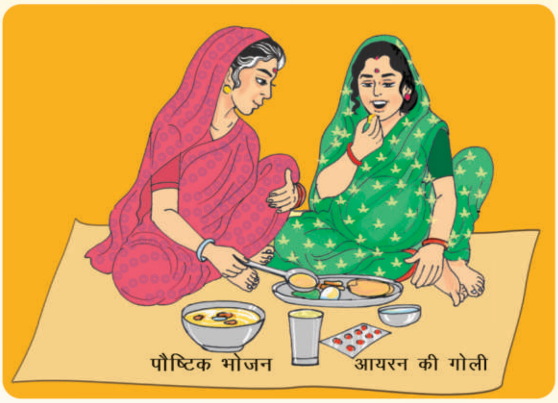

तालिका–1

क्र.सं	विभिन्न प्रकार के खाद्य समूह	खाद्य समूह के अंतर्गत आने वाले खाद्य पदार्थ	विभिन्न प्रकार के खाद्य समूह से मिलने वाले लाभ
1	अनाज, कन्द और मूल	चावल, गेंहू , बाजरा, जवार आलू , शकरकन्द	हमे दैनिक कार्यों को करने की उर्जा व शक्ति मिलती है।
2	दालें, फलियाँ एवं मेवा	चना , मूंग, अरहर,मसूर दाल , मटर , छोले, राजमा	हमारे शारीरिक वृधि एवं विकास अच्छा होता है।
3	दूध एवं दुग्ध पदार्थ	दूध, दही, मट्ठा, छाछ, पनीर	मानसिक विकास तेजी से होती है व हड्डियाँ भी मजबूत होती है।
4	मांसाहारी भोजन	मांस, मछली, कलेजी	मांसपेशियों को मजबूत बनाता है।
5	विटामिन युक्त फल एवं सब्जियां (नारंगी, पीले रंग की फल एवं सब्जियां)	पपीता, आम, संतरे, कद्दू, गाजर	रोग प्रतिरोधक क्षमता को बढ़ाते है मानसिक विकास तेजी से होता है , रतौंधीसे बचाता है।
6	अन्य फल एवं सब्जियां (हरी पत्तेदार सब्जियां एवं अन्य फल)	पालक, सरसो का साग, बथुआ, मेथी, लौकी,सेम, तुरोयी, करेला	शरीर में खून की वृद्धिकरता है व बिमारियों से बचाव में सहायता करता है।
7	अंडे	अंडे	मांसपेशियों व् हड्डियों को मजबूत बनाता है।

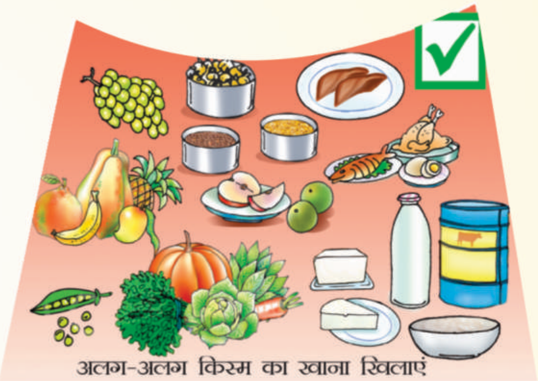

- गर्भावस्था के दौरान पहले से एक बार अधिक भोजन थोड़े–थोड़े अन्तराल पर करें (कम से कम 3 बार) और पहले से कुल डेढ़ गुना मात्रा मे भोजन करें, और साथ ही दो बार नाश्ता भी करें, इससे गर्भ में पल रहे बच्चे का उचित विकास होगा। धात्री महिला पहले से दोगुनी मात्रा में आहार लें।
- खाद्य समूह एवं मात्रा के बारे में तालिका के अनुसार परामर्श दें और एक पाव (250 मिली) के कटोरे से मात्रा का प्रदर्शन करें।

गर्भवती महिला के लिए दैनिक भोजन चार्ट/मात्रा

खाद्य समूह	मात्रा (250 ml की कटोरी)
गाढ़ी दाल	2 कटोरी *
रोटी/चावल	6–8 पीस/4 कटोरी
गहरी हरी पत्तेदार सब्जी	1 कटोरी
नारंगी/पीले रंग की फल एवं सब्जी	1 कटोरी
दूध अथवा दूध से बने पदार्थ	1 ग्लास/1 कटोरी
**मांसाहारी महिलायें अपने पसन्द के अनुसार निम्न चीजें दैनिक भोजन में शामिल कर सकती हैं**	
अंडा	1 पीस
मांस/मछली/कलेजी	1 पीस (माचिस की डिब्बी के बराबर)
**दैनिक आहार के अलावा गर्भवती महिला पौष्टिक नाश्ता भी लें**	
पौष्टिक नाश्ता	2 बार

*1 कटोरी = 250 ml

(गर्भवती महिला पहली तिमाही के बाद अपने भोजन की मात्रा बढ़ा कर खायें)

– **गर्भावस्था में वजन वृद्धि को मापें–**

- गर्भावस्था के दौरान 9 से 11 कि.ग्रा. वजन बढ़ता है। (हर महीने औसतन 1.5 कि0ग्रा0 से 2 कि0ग्रा0 वजन बढ़ता है)
- हर माह ग्राम स्वास्थ्य एवं पोषण दिवस पर वजन वृद्धि की माप करायें।

– **गर्भावस्था में आयरन की गोली का सेवनः**

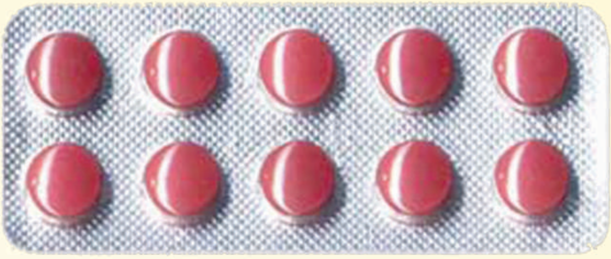

आयरन की गोलियाँ

- पहली तिमाही के बाद 1 गोली प्रतिदिन की दर से 180 गोलियाँ आयरन की लेनी चाहिये। एनीमिया (HB<11gm) होने पर 2 गोली प्रतिदिन लेनी चाहिऐ।[1]
- आयरन की गोली जहाँ तक सम्भव हो खाना खाने के एक घण्टे बाद या रात में लेनी चाहिये।[2] ऐसा करने से आयरन की गोली के पार्श्व प्रभावों जैसे उल्टी आना, जी मिचलाना, चक्कर आना, सर भारी होना इत्यादि से बचा जा सकता है।
- गर्भावस्था के दौरान आयरन की गोली का सेवन बच्चों के बढ़ने और उनके मस्तिष्क के विकास में मदद करता है। गर्भावस्था में आयरन की कमी होने पर माँ को एनीमिया हो सकता है, माँ व बच्चे दोनों को खतरा होने की सम्भावना रहती है,जैसे कि प्रसव उपरान्त माँ को अत्यधिक रक्तश्राव होना एवं बच्चे का जन्म के समय कम वजन का होना इत्यादि।
- गम्भीर एनीमिया (HB<7gm) होने पर महिला को स्वास्थ्य केन्द्र पर संदर्भित करें जहाँ पर महिला को डाक्टर की सलाह पर आयरन का इंजेक्शन लगेगा या खून चढ़ाया जायेगा।

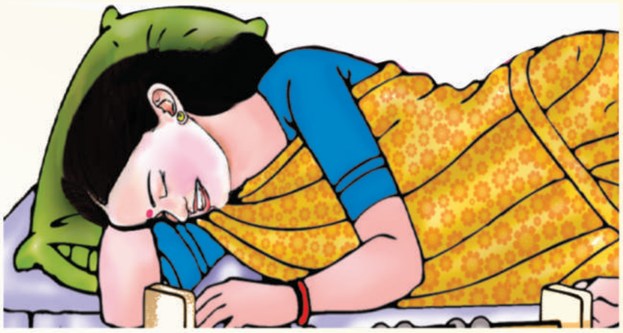

गर्भावस्था में आराम

– **आंगनवांड़ी केंद्र पर मिलने वाले पूरक आहार का उपभोग केवल गर्भवती एवं धात्री द्वारा किया जाना चाहिये, पूरे परिवार द्वारा नहीं।**

– **गर्भावस्था में निम्न बातों का ध्यान रखना चाहिएः**

- रात में कम से कम 8 घंटे और दिन में 2 घंटे की नींद लें। कोई भी भारी वस्तु नहीं उठायें या कोई भी कठिन मेहनत न करें।
- परिवार को गर्भवती महिला के दैनिक कार्यों में सहायता करें।

❊ **स्तनपान कब आरम्भ करना चाहिए, केवल स्तनपान कब तक कराना चाहिए?**

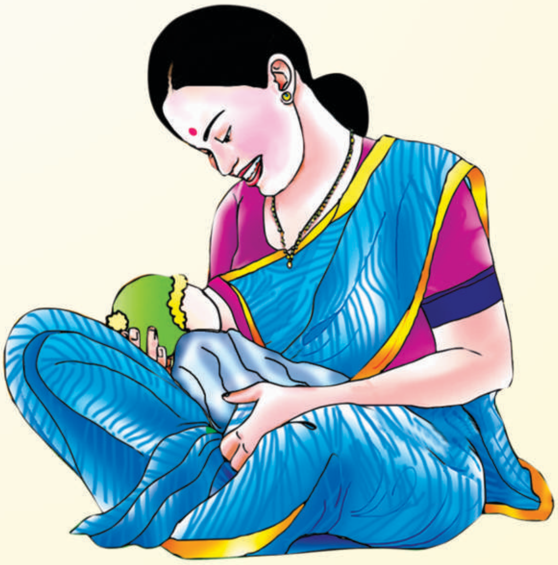

स्तनपान

• **संदेशः**

– **गर्भवती महिला के लिये स्तनपान सम्बन्धी जानकरीः**

- जन्म के तुरन्त बाद नवजात शिशु को एक घन्टे के अंदर स्तनपान करायें। इससे दूध भी जल्दी उतरता है।
- शिशु को छः माह तक केवल माँ का दूध दिया जाना चाहिये (पानी भी नहीं)।

1. नेशनल आयरन प्लस इनीशिएटिव (NIPI),परिवार कल्याण, उ0प्र0, 2015।
2. गाइड लाइन फार कन्ट्रोल ऑफ आयरन डेफिशिएन्सी एनीमिया, स्वास्थ्य एवं परिवार कल्याण मंत्रालय, भारत सरकार, 2013।

अध्याय–2

0–6 माह तक के बच्चों में शीघ्र व केवल स्तनपान

जन्म के तुरन्त बाद स्तनपान व 6 माह तक केवल माँ का दूध देने से शिशु को सभी पोषक तत्व मिलतें हैं और शिशु बीमारियों से बचा रहता है एवं उसका विकास सही होता है।

✦ **समूह चर्चा के दौरान संवाद शुरू करने के लिए पूछे जाने वाले प्रश्न तथा उनसे सम्बंधित संदेशः**

❊ **स्तनपान कब आरम्भ करना चाहिए और कब तक केवल स्तनपान कराना चाहिए?**

★ **संदेशः**

- जन्म के तुरन्त बाद नवजात शिशु को एक घण्टे के अंदर स्तनपान करायें। इससे दूध भी जल्दी उतरता है, एवं माँ के दूध की निरन्तरता बनी रहती है व शिशु को उचित पोषण भी मिलता है।
- शिशु को छः माह तक केवल माँ का दूध दें (पानी भी नहीं) माँ के दूध में शिशु की आवश्यकता अनुसार पानी होता है।

❊ **क्या बच्चे को माँ के दूध के अलावा कुछ और भी देना चाहिए?**

★ **संदेशः**

- शिशु को पानी, घुटी,शहद, चीनी का पानी, गाय या भैंस का दूध आदि कुछ भी नहीं पिलायें। केवल माँ का पहला पीला गाढ़ा दूध (खीस) जरूर दें, यह बच्चे में रोग प्रतिरोधक क्षमता को बढ़ाता है व किसी भी प्रकार के संक्रमण से बचाव करता है।
- डॉक्टर के निर्देशानुसार आवश्यकता पड़ने पर ओ0 आर0 एस0, वैक्सीन, विटामिन व मिनरल का सिरप दे सकते हैं।

❊ **माँ और बच्चे को स्तनपान से क्या लाभ है?**

★ **संदेशः**

– **स्तनपान से शिशु को लाभः**

- माँ का दूध 6 माह तक शिशु के मानसिक एवं शारीरिक विकास के लिए सम्पूर्ण आहार है।
- माँ का दूध शिशु को रोगों से बचाता है
- शिशु को माँ के शरीर से गर्माहट मिलती है।
- माता और शिशु में घनिष्ठ और स्नेहपूर्ण सम्बन्ध बनता है।

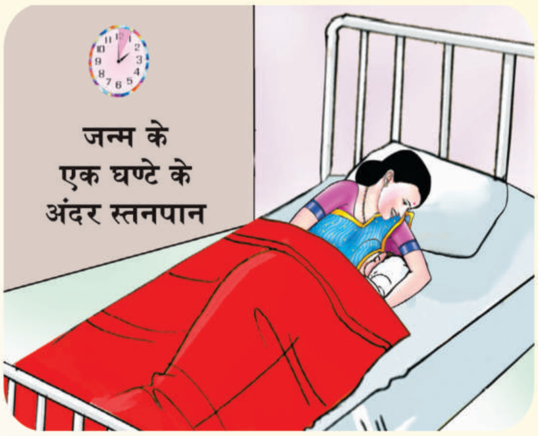

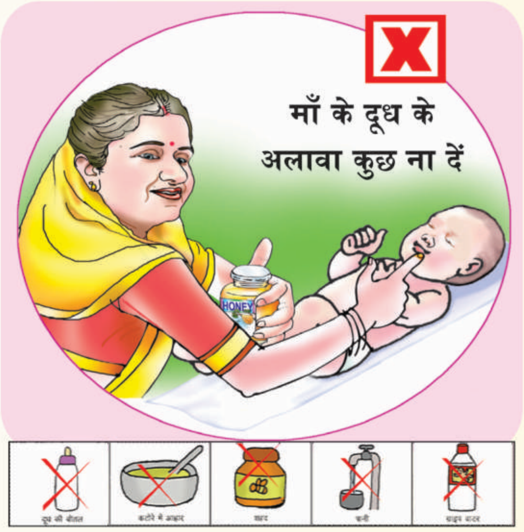

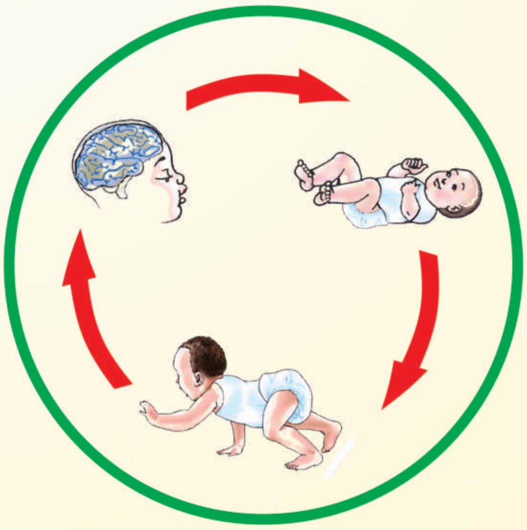

स्तनपान से मानसिक और शारीरिक विकास अच्छा होता है।

– स्तनपान कराने से माँ को लाभः

- जन्म के तुरंत बाद स्तनपान से गर्भाशय सिकुड़ने में सहायता मिलती है।
- प्लेसेंटा (आंवल) जल्द बाहर आता है।
- प्रसव के बाद अधिक खून बहने का खतरा घट जाता है।

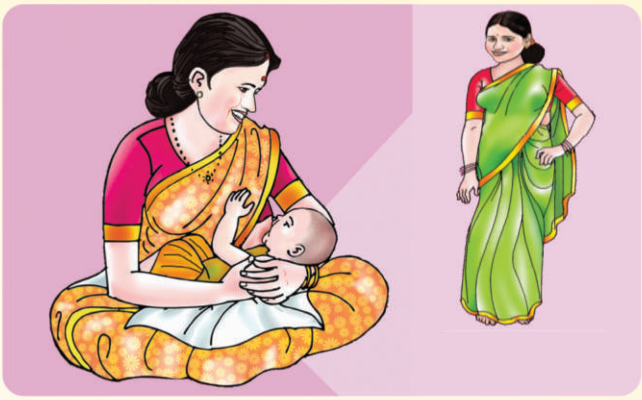

स्तनपान के दौरान निम्न बातों का ध्यान रखें।

– दूध पिलाते समय माँ व बच्चे की अवस्था क्या होनी चाहिए?

- माँ आराम से अपनी पीठ को सहारा देते हुए बैठे तथा शिशु को कमर एवं निचले भाग से सहारा देकर पकड़े।
- शिशु का शरीर एवं चेहरा माँ शरीर की तरफ मुड़ा हो।
- दूध पीते समय बच्चे का निचला ओंठ नीचे की ओर झुका हुआ हो तथा मुँह बड़ा खुला हुआ हो एवं नाक माँ के स्तन को छू रही हो। माँ के स्तन का अधिकतर भूरा भाग बच्चे के मूँह के अन्दर हो तथा बच्चे के दूध गटकने की आवाज सुनाई दे।

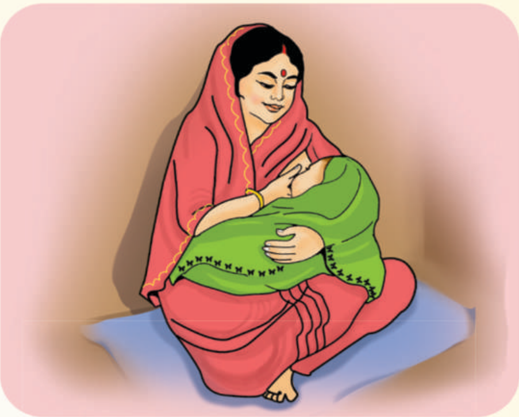

सही अवस्था (बैठे हुये)

– माँ का दूध शिशु के लिए पर्याप्त है इसका पता कैसे करें?

- शिशु 24 घन्टे में कम से कम 6 बार पेशाब करता हो।
- शिशु ठीक से सोता एवं खेलता हो।
- शिशु का वजन बढ़ रहा हो।

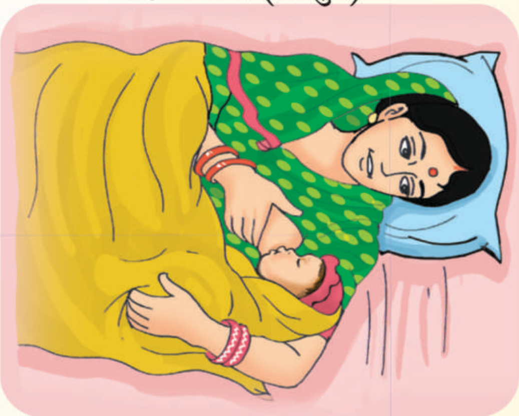

सही अवस्था (लेटे हुये)

स्तनपान के दौरान माँ के दूध की पर्याप्त मात्रा बनाये रखनाः

- आवश्यक है कि माँ द्वारा शिशु को बार–बार दूध पिलाया जाना चाहिये, जब भी बच्चा चाहे, दिन और रात दोनों समय।
- माँ के दूध के अलावा शिशु को ऊपर से कुछ भी नहीं दिया जाना चाहिये, पानी भी नहीं।

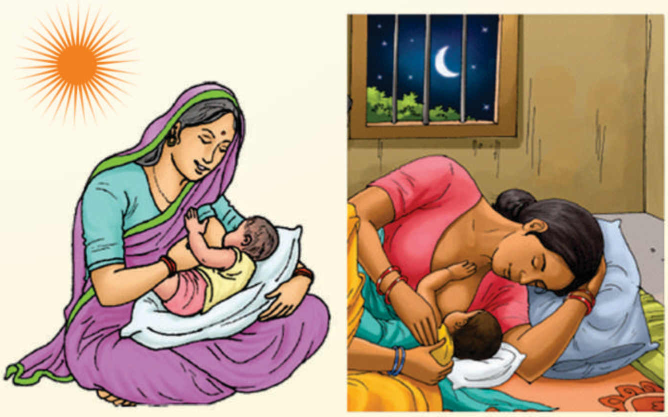

स्तनपान दिन और रात दोनों समय

अध्याय–3

6 माह से 2 साल के बच्चों में पूरक आहार

बच्चे के 6 माह पूरा हो जाने के बाद माँ का दूध उसके पोषण के लिए पर्याप्त नहीं होता, उसे माँ के दूध के साथ पूरक आहार देना भी आवश्यक हो जाता है।

✦ **समूह चर्चा के दौरान संवाद शुरू करने के लिए पूछे जाने वाले प्रश्न तथा उनसे सम्बंधित संदेश:**

(चर्चा से पूर्व दैनिक खान–पान के बारे में जानकारी ले तथा उपलब्ध स्थानिक भोजन सामग्री के अनुसार सलाह दें)

❈ **बच्चे को पूरक आहार कब शुरू करना चाहिये और क्यों?**

★ **संदेश:**

- बच्चे के 6 माह (180 दिन) पूरा होने पर पूरक आहार देना शुरू करें, ऐसा इसलिए करते हैं क्योंकि 6 माह के बाद बच्चा तीव्रता से बढ़ता है तथा उसे अतिरिक्त पोषण की आवश्यकता होती है जो केवल स्तनपान से पूर्ण नहीं होती है।
- 7 खाद्य समूह (अनाज, कन्द और मूल, दालें, फलियाँ एवं मेवा, दूध एवं दुग्ध उत्पाद, मांसाहारी भोजन, अण्डे, विटामिन–ए युक्त फल व सब्जियां (नारंगी/पीले रंग के फल एवं सब्जियाँ जैसे कि गाजर,पपीता,आम इत्यादि) और अन्य फल व सब्जियां) में से 4 खाद्य समूह दैनिक आहार में शामिल करें।[1]

(इन खाद्य समूहो की विस्तृत जानकारी के लिए अध्याय–1 में दी गई तालिका नं0 1 को पढ़ें)

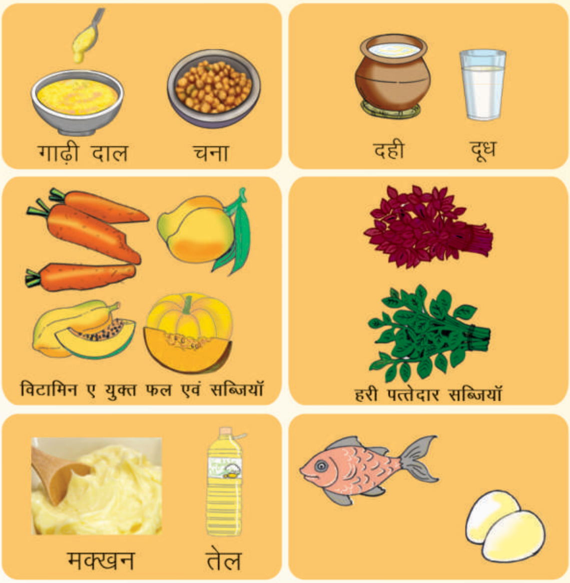

विभिन्न खाद्य समूह

❈ **आयु के अनुसार पूरक आहार देने के दौरान कौन–कौन सी बातों का ध्यान रखना चाहिये?**

★ **संदेश:**

– 6 से 8 माह के शिशु के लिए

- घर का बना हुआ ठोस/अर्द्धठोस आहार मसल कर देना चाहिए, तरल भोजन न दें।

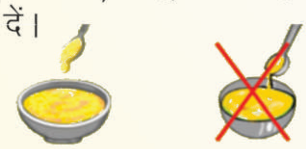

- अंडा, दही, दलिया, गाढ़ी दाल, गहरी हरी पत्तेदार सब्जियां या पीले फल दें।
- दिन में दो बार आधी–आधी कटोरी दें।

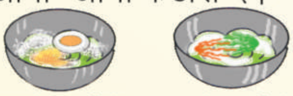

- अतिरिक्त ऊर्जा के लिए एक चम्मच अतिरिक्त घी/तेल भोजन में डालें।

– 9 से 11 माह शिशु के लिए:

- शिशु को समय देना चाहिये एवं स्वयं भोजन करने की आदत डालनी चाहिये।
- अंडा, दही, दलिया, गाढ़ी दाल, गहरी हरी पत्तेदार सब्जियां या पीले फल दें।

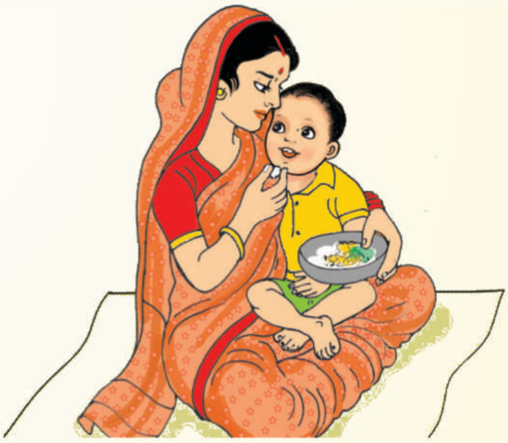

बच्चे को स्नेहपूर्वक खिलायें

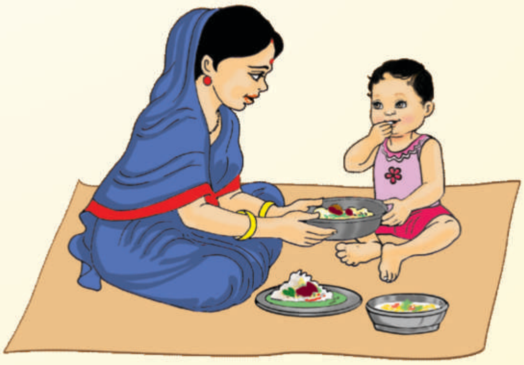

बच्चे को स्वयं खाना उठाकर खाने की आदत डालें।

1. आई0वाई0सी0एफ0 गाइड लाइन, स्वास्थ्य एवं परिवार कल्याण मंत्रालय, भारत सरकार, 2013।

- दिन में 3 बार आधी कटोरी एवं एक से दो बार पौष्टिक नाश्ता (पका हुआ पपीता, पका आम, दही उबला अंडा, आंगनवाड़ी में मिलने वाला पुष्टाहार) दें।

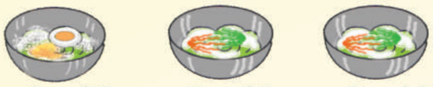

– 12 से 23 माह के शिशु के लिए

- शिशु को स्वयं अलग थाली या कटोरी में भोजन करने के लिए प्रोत्साहित करें।
- अंडा, दही, गाढ़ी दाल, गहरी हरे पत्तेदार सब्जियाँ एवं पीले फल दें ।
- दिन में 3 बार पूरी कटोरी एवं एक–दो बार पौष्टिक नाश्ता (पका हुआ पपीता, आम, दही, उबला अंडा, आंगनवाड़ी में मिलने वाला पुष्ठाहार दूध से बनी चीजें) दें।

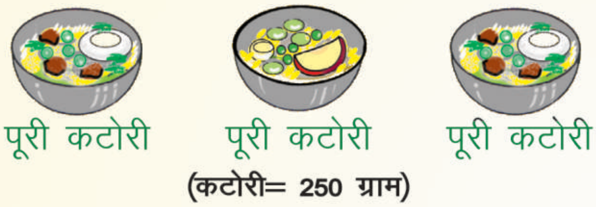

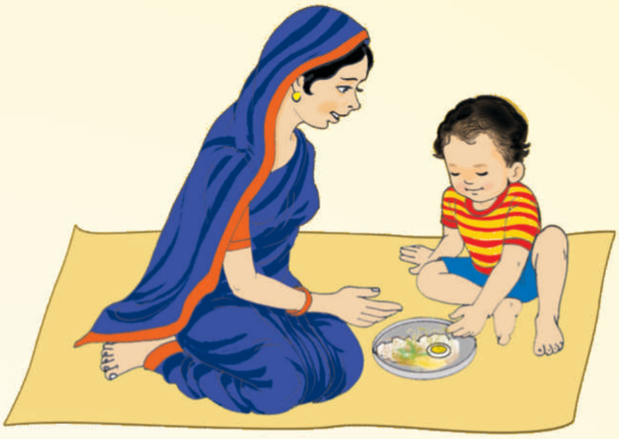

शिशु को स्वयं भोजन करने के लिए प्रोत्साहित करें।

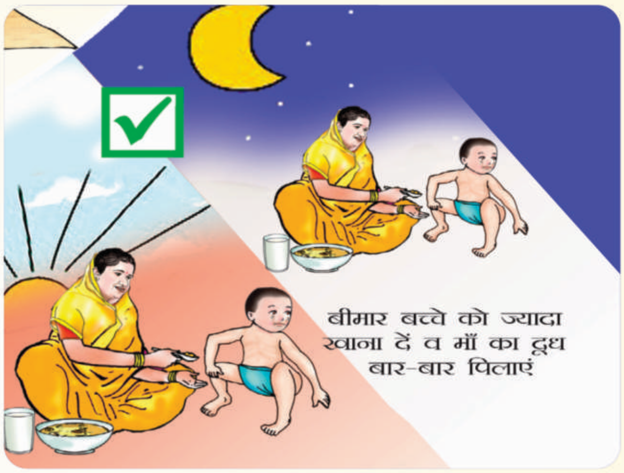

❈ बीमार शिशु के भोजन के बारे में किन–किन बातों का ध्यान रख्ना चाहिए?

★ **संदेशः**

- बीमार शिशु को भोजन कराना कम या बन्द नही करना चाहिए व बार–बार स्तनपान कराते रहें।
- शिशु की पसंद के अनुरूप आहार दें।
- बीमारी के दौरान बच्चा कमजोर हो जाता है, ठीक होने के उपरान्त उसके खाने की मात्रा बढ़ा दें, जब तक कि शिशु का वजन पहले के बराबर न हो जाए।

– शिशु को पूरक आहार खिलाने समय निम्न बातों का ध्यान रखें

★ अनिच्छा से खाने वाले(कम खाने वाले) शिशु को खिलाना–

- जब शिशु को भूख लगे तभी भोजन कराएँ।
- भोजन को बदल–बदल कर दें।
- शिशु को पानी, जूस, चाकलेट, चिप्स कुरकुरे, शर्बत इत्यादि से पेट नहीं भरने दें।
- प्रत्येक कौर खाने पर शिशु को प्रोत्साहित करें।
- शिशु को समय दें एवं शिशु को भोजन कराते समय धैर्य रखें व खाना खिलाते समय कभी भी ज़बरदस्ती न करें।

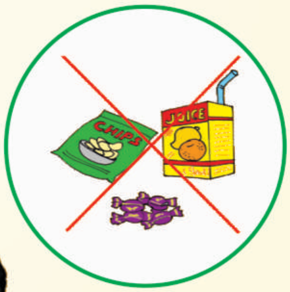

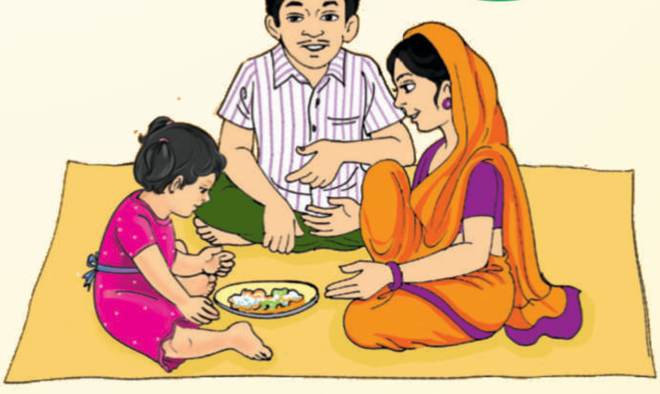

कम खाने वाले शिशु को खिलाना

अध्याय–4

बच्चों में सूक्ष्म पोषक तत्वों का सम्पूरण
(आयरन, विटामिन–ए, आयोडीन, ज़िक–ओ. आर. एस. एवं डिवार्मिंग)

आहार के अलावा सूक्ष्म पोषक तत्वों के सम्पूरण से बच्चों में रोग प्रतिरोधक क्षमता बढ़ती है साथ ही उचित मानसिक व शारीरिक विकास होता है।

✦ **समूह चर्चा के दौरान संवाद शुरू करने के लिए पूछे जाने वाले प्रश्न तथा उनसे सम्बंधित संदेशः**

❊ **क्या आप जानते है कि बच्चों को आयरन सिरप कब और क्यों देना चाहिये?**

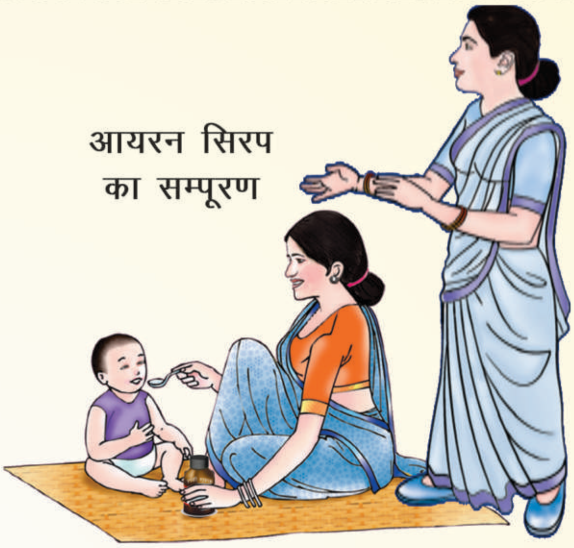

आयरन सिरप का सम्पूरण

★ **संदेशः**

– **आयरन सिरप का सम्पूरणः**

- इससे बच्चों में खून की कमी को रोका जा सकता है।
- 6 माह का होते ही शिशु को आयरन सिरप पिलाना आरम्भ करें, यह ए0एन0एम0 से प्राप्त किया जा सकता है।
- 6 माह से 5 वर्ष तक सभी बच्चों को 1-1ml हफ्ते में दो बार आयरन सिरप दिया जाना है।

❊ **क्या आप जानते है कि बच्चों को विटामिन–ए कब और क्यों देना चाहिये?**

★ **संदेशः**

– **विटामिन–ए का सम्पूरणः**

- 9 माह का होते ही बच्चे को विटामिन–ए सिरप पिलाया जाता है। विटामिन–ए से बच्चे में रोग–प्रतिरोधक क्षमता बढ़ती है तथा आँख से सम्बन्धित रोगों से बचाता है।
- 9 माह से 5 वर्ष तक सभी बच्चों को, साल में दो बार (6–6 माह के अंतराल पर कुल नौ बार) विटामिन 'ए' दें।
- पहली खुराक मिज़ल्स के टीके व दूसरी खुराक मिजल्स बूस्टर के साथ डेढ़ साल पर VHND के दौरान दी जाती है और फिर 3 से 9वीं खुराक 6–6 माह के अन्तराल पर (जून और दिसम्बर माह में) बाल स्वास्थ्य पोषण माह के दौरान ANM द्वारा दी जाती है।
- जच्चा बच्चा कार्ड पर विटामिन–ए की सभी खुराखों को दर्ज करवाऐं।

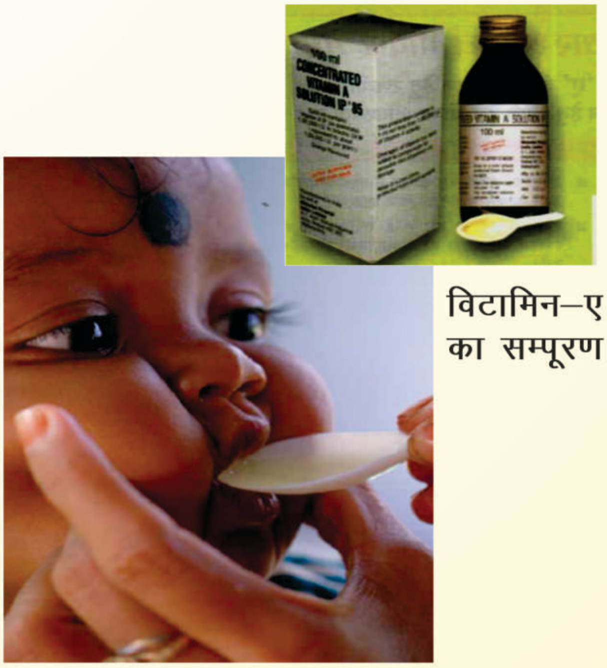

विटामिन–ए का सम्पूरण

❊ **क्या आप जानते है कि आयोडीन युक्त नमक को सेवन क्यों करना चाहिये?**

★ **संदेश:**

– आयोडीन युक्त नमक का सेवन:

- पूरा परिवार घर पर खाने में आयोडीन युक्त नमक का ही प्रयोग करें।
- इससे घेंघा जैसे रोगों से बचाव होता है तथा बच्चें का मानसिक विकास सही से होता है।

शक्ति और बुद्धि का रक्षक

आयोडीन नमक

❊ **क्या आप जानते है कि ओ. आर. एस. एवं जिंक का प्रयोग कब और कैसे करना चाहिये?**

★ **संदेश:**

– ओ. आर. एस. एवं जिंक:

- ओ. आर. एस. एवं जिंक का प्रयोग बच्चों में दस्त की रोकथाम के लिए किया जाता है। बार–बार दस्त होने से बच्चा कुपोषित हो जाता है।

- दस्त में दो से छः माह तक के बच्चों में माँ का दूध जारी रखें और साथ ही ओ. आर. एस. का घोल तब तक पिलायें जब तक बच्चे का दस्त ना रुके तथा जिंक की आधी–आधी गोली 14 दिनों तक लगातार दें।

- दस्त में छः माह से पांच वर्ष तक के बच्चों में स्तनपान व भोजन जारी रखें और साथ ही ओ. आर. एस. का घोल तब तक पिलायें जब तक बच्चे का दस्त ना रुके तथा जिंक की एक–एक गोली 14 दिनों तक लगातार दें।

- ओ0आर0एस0 एवं जिंक की गोलियॅा ए0एन0एम0 एवं आशा से प्राप्त करें।

ओ.आर.एस. घोल बनाने का तरीका

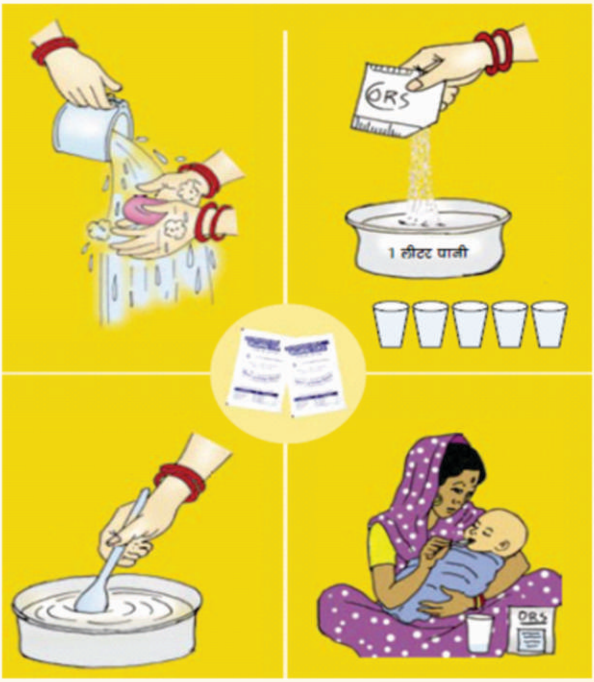

– ओ.आर.एस. घोल बनाने का तरीका:

- साफ बर्तन में एक पैकेट ओ.आर.एस. डालें और एक लीटर पीने का साफ पानी लें और ओ.आर.एस. पाउडर का पूरा पैकेट अच्छी तरह पानी में घोल लें। बच्चे को ओ.आर.एस. घोल चम्मच या कप में डाल कर थोड़ी–थोड़ी देर में पिलाएं। **24 घंटे बाद यदि ओ.आर.एस. घोल बच जाये तो उसे फेंक दें।**

❊ **बच्चों मे कृमि रोकथाम (डिवार्मिंग) के लिए क्या करें?**

- राष्ट्रीय कृमिनाशक कार्यक्रम के अन्तर्गत बच्चों में कृमि से रोकथाम के लिए साल में दो बार छः–छः माह के अन्तराल पर एल्बेंडाजोल की गोली या कृमिनाशक अवश्य दें।

अध्याय–5

वृद्धि निगरानी

नियमित वृद्धि निगरानी बच्चों के पोषण स्तर के आकलन व समय से उचित कदम उठाने में मदद करती हैं।

✦ **समूह चर्चा के दौरान संवाद शुरू करने के लिए पूछे जाने वाले प्रश्न तथा उनसे सम्बंधित संदेश:**

❊ **बच्चों में वृद्धि निगरानी क्या है? और वृद्धि निगरानी क्यों आवश्यक है?**

★ **संदेश:**

- 0 से 3 वर्ष के बच्चों में मासिक एवं 3 से 5 वर्ष तक के बच्चों के लिये तिमाही वजन आंगनवाड़ी केन्द पर लिया जाता है, वजन लेकर जब वृद्धि चार्ट पर बच्चे की आयु के समकक्ष प्लाट किया जाता है तो बच्चे की वृद्धि का पता चलता है। लडको के लिए नीले रंग का एवं लड़कियों के लिऐ गुलाबी रंग के चार्ट का प्रयोग किया जाता हैं।
- 0 से 3 वर्ष के बच्चे का वजन जच्चा बच्चा कार्ड (MCP) में भी अंकित करवायें।

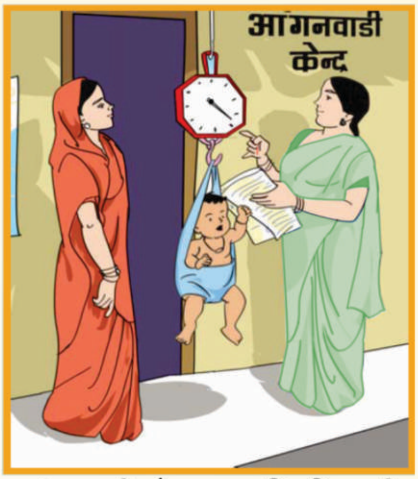

आंगनवाड़ी केन्द्र पर वृद्धि निगरानी

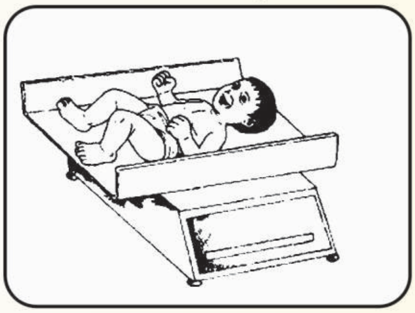

❊ **बच्चे के पीले और लाल श्रेणी में होने पर क्या करें?**

- बच्चे के कुपोषण का पता चलने पर आंगनवाड़ी कार्यकत्री के पास ले जायें एवे आयु के अनुसार उचित परामर्श और सेवाएं पायें।
- बच्चा अगर लम्बे समय तक पीले श्रेणी में है तो उसे नजदीकी स्वास्थ्य केंद्र पर स्वास्थ्य जाँच के लिये ले जायें।
- बच्चा अगर लाल श्रेणी में है तो बच्चे को आंगनवाड़ी से दोगुना राशन प्राप्त करें, उचित आहार सम्बन्धित परामर्श लें, एवं उसे नजदीकी स्वास्थ्य केन्द्र पर परीक्षण हेतु लेकर जायें ।
- किसी प्रकार की चिकित्सीय जटिलता मिलने पर भी तुरंत उसे नजदीकी स्वास्थ्य केंद्र पर लेकर जायें।
- डाक्टरी सलाह के अनुसार यदि बच्चा गम्भीर कुपोषित की श्रेणी में आता है तो उसे जिला अस्पताल स्थित पोषण पुनर्वास केन्द्र पर ले जायें।

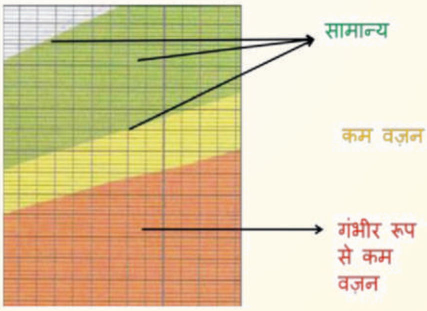

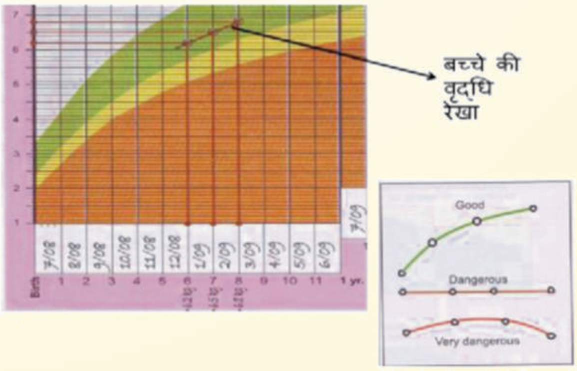

ग्रोथ चार्ट में रंग एवं रेखा के घुमाव को देखकर बच्चे के पोषण की स्थिति का पता लगाया जाता हैं।

★ पोषण पुनर्वास केन्द्रः

पोषण पुनर्वास केन्द्र को गम्भीर कुपोषित बच्चों के उपचार एवं देखभाल के लिए ज़िला अस्पताल में बनाया गया है, जिसमें बच्चों को कुपोषण से मुक्त कराने के लिए उचित चिकित्सीय सहायता एवं परामर्श प्रदान किया जाता है।

– 6 माह से 59 माह के बच्चों के भर्ती के मानक

- बच्चे की लम्बाई में वजन–3SD से कम।
- बच्चे की मिड अपर का माप 11.5 सेमी0 से कम।
- बच्चे के दोनों पैरों में पिटिंग एडीमा।

✦ गम्भीर कुपोषित बच्चों में कभी–कभी अन्य जटिलतायें भी हो सकती हैं उन्हें प्राथमिकता के अनुसार उपचार हेतु भर्ती किया जाये।

– 6 माह से छोटे बच्चों के भर्ती के मानक

- बच्चे की लम्बाई के अनुपात में वजन–3SD से कम (यदि शिशु की लम्बाई 45 सेमी0 से अधिक) है।
- बच्चे के दोनों पैरों में पिटिंग एडीमा।
- देखने में अति गम्भीर कुपोषित।

✦ गम्भीर कुपोषित बच्चों में कभी–कभी अन्य जटिलतायें भी हो सकती हैं उन्हें प्राथमिकता के अनुसार उपचार हेतु भर्ती किया जाये।

– पोषण पुनर्वास केन्द्र से छुट्टी के मानक

- बच्चे के वजन में 15 प्रतिशत की वृद्धि होना।
- बच्चे के वजन में कम से कम 5 ग्राम/किलोग्राम /दिन की वृद्धि लगातार तीन दिन तक हो रही हो।
- बच्चे की भूख का वापस आना एवं वह उचित मात्रा में पौष्टिक भोजन ले रहा है।
- बच्चे के शरीर पर सूजन न होना।
- बच्चे के संक्रमण व बीमारी के उपचार होने पर

स्वच्छता एवं साफ सफाई

बच्चों में कुपोषण का एक प्रमुख कारण स्वच्छता एवं साफ सफाई की कमी भी हो सकती है।

✦ **समूह चर्चा के दौरान संवाद शुरू करने के लिए पूछे जाने वाले प्रश्न तथा उनसे सम्बंधित संदेशः**

❊ **स्वच्छता एवं साफ सफाई का ध्यान रखना क्यों ज़रुरी है?**

★ **संदेशः**

- संक्रमित हाथ, संक्रमित मक्खी, संक्रमित जल व जमीन व मिटटी में कीटाणु (bacteria, virus आदि) पाये जाते हैं। यह कीटाणु अंततः संक्रमित भोजन (5F- fingers, flies, food, fluid and field) के माध्यम से शरीर में प्रवेश करते हैं।
- अस्वच्छ वातावरण व गंदगी का लघु कालीन प्रभाव– बच्चों में डायरिया व दस्त का बार बार होना।
- बच्चे का विकास 6 माह के बाद बहुत तेजी से होता है और उसे मां के दूध के साथ उपरी आहार से उर्जा प्राप्त करना आवश्यक हो जाता है। इसी समय उसकी बीमारियों से लडने की क्षमता का भी विकास होता है।
- डायरिया व संक्रमण के कारण विकास हेतु आवश्यक उर्जा का इस्तेमाल संक्रमण से बचाव के लिये होने लगता है। बच्चे की वृद्वि की गति इस प्रकार धीमी पड़ जाती है। साथ–साथ यदि वातावरण लम्बे समय तक दूषित रहता है तो पाचन प्रक्रिया भी सुस्त होती जाती है जिससे बच्चा जो कुछ भी खाता है वह शरीर ग्रहण नही कर पाता है।
- संक्रमण लम्बे समय तक रहता है तो विकास की गति धीमी होती जाती है और बच्चा नाटा रह जाता है।

ध्यान रहे– शिशु मल व्यस्क मल से अधिक दूषित होता है और उसमें कीटाणु भी अधिक होते हैं।

❊ **स्वच्छता व्यवहार कौन–कौन से हैं ?**

★ **संदेश**

- शिशु को भोजन खिलाने से पूर्व शिशु व माँ को, शिशु को स्तनपान कराने से पूर्व एवं शिशु का मल साफ करने के बाद साबुन से हाथ धोना चाहिए।
- शौच के बाद, भोजन करने से पूर्व, भोजन बनाने से पूर्व, नाक साफ करने के बाद एवं छींकने के बाद हाथ साबुन से धोना चाहिए।
- भोजन व पानी को सदैव ढक कर रखना चाहिये तथा पानी निकालने के लिए डंडीदार लोटे का उपयोग करना चाहिए। ताजा भोजन बनायें एवं बासी भोजन का प्रयोग नहीं करना चाहिए।
- पानी उबाल कर पीने से पानी में मौजूद सभी कीटाणु नष्ट हो जाते हैं तथा पानी पीने योग्य हो जाता है।
- खुले में शौच नहीं करना चाहिए क्योंकि इसमें विभिन्न प्रकार की बीमारी पैदा करने वाले कीटाणु वातावरण में फैल जाते हैं तथा खाने व् पेयजल को दूषित करते हैं, इसलिए घर में शौचालय बनवाएं एवं उसका नियमित प्रयोग करें।
- बच्चे के मल को साफ करने के बाद सुरक्षित ढंग से निस्तारण करें व इधर–उधर ना फेंकें ।

मल का मुँह तक संचरण का मार्ग (5-F)

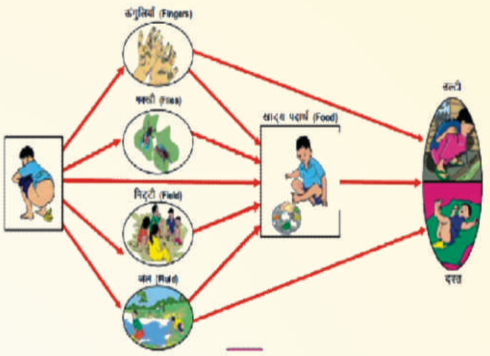

खाना बनाने और खिलाने से पहले, साबुन से हाथ अवश्य धोएँ

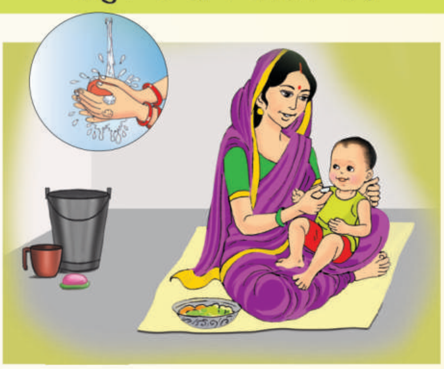

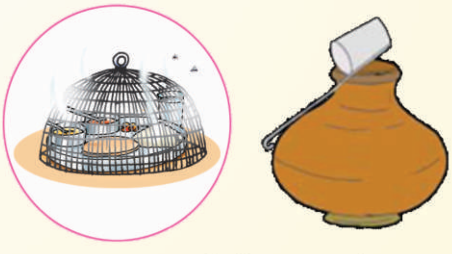

खाना एवं पानी को ढक कर रखे

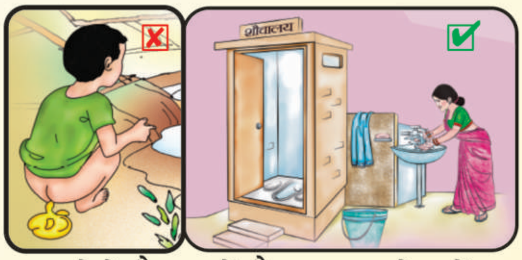

खुले मे शौच न करें शौचालय का प्रयोग करें

अध्याय–7

प्रजनन स्वास्थ्य

किशोरावस्था में स्वास्थ्य एवं पोषण व विवाह के बाद समय पर परिवार नियोजन का ध्यान रखने से महिला स्वस्थ रहती है।

✦ **समूह चर्चा के दौरान संवाद शुरू करने के लिए पूछे जाने वाले प्रश्न तथा उनसे सम्बंधित संदेश:**

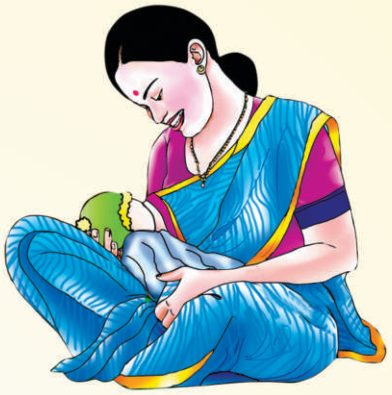

❊ **बार–बार गर्भधारण से माँ के स्वास्थ्य पर क्या प्रतिकूल प्रभाव पड़ता है, व परिवार नियोजन के क्या लाभ है?**

★ **संदेश:**

- कम अन्तराल पर एवं बार –बार गर्भधारण से माँ शारीरिक रुप से कमजोर हो जाती है जिस के कारण होने वाला बच्चा कमजोर पैदा होता है।
- दोबारा गर्भवती होने के लिये माँ के शरीर को पूरी तरह से तैयार होने में कम से कम तीन साल का समय लग जाता है, ऐसे में परिवार नियोजन एक उचित उपाय है।
- परिवार नियोजन से माँ एवं शिशु दोनो स्वस्थ रहते हैं एवं कुपोषण से भी बचाव होता है।
- परिवार नियोजन की जिम्मेदारी महिला के साथ–साथ पुरुष की भी होती है

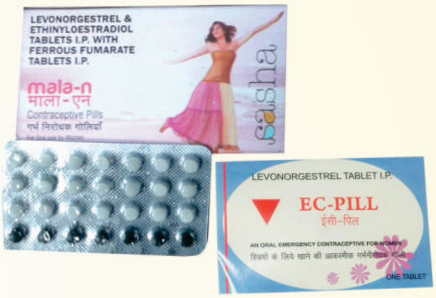

❊ **शादी के बाद दो साल तक एवं दो बच्चों के बीच 3 साल का अन्तराल रखने के लिए किन–किन विधियों को अपनाया जा सकता है?**

★ **संदेश:**

- माँ यदि जन्म से 6 माह तक शिशु को केवल स्तनपान कराती है तो इस दौरान पुनः गर्भधारण की सम्भावना कम होती है। इसमें निम्न बातों का ध्यान रखना अति आवश्यक है–
  1. छः माह तक केवल स्तनपान।
  2. माहवारी दुबारा न आयी हो।

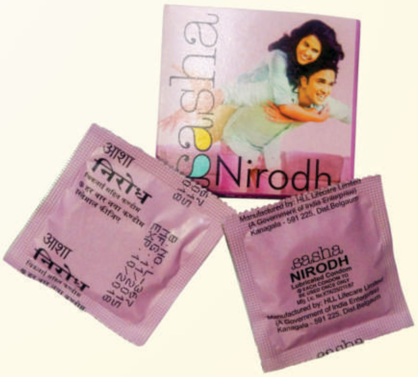

– परिवार नियोजन के तरीके:

(बच्चों के जन्म के अन्तराल हेतु निम्न साधन अपनाये जा सकते हैं।)

- कंडोम
- गर्भ निरोधक गोलियां (डॉक्टरी सलाह के उपरान्त)
- आकस्मिक गर्भ निरोधक गोलियां (ECP) (डॉक्टरी सलाह के उपरान्त)
- कॉपर–टी (अन्तराल विधि)
- प्रसव पश्चात कॉपर–टी।
- DMPA इंजेक्शन(गर्भ निरोधक टीकें)
- परिवार नियोजन के सभी साधन और सलाह सरकारी स्वास्थ्य केन्द्रों पर निःशुल्क उपलब्ध होती है।

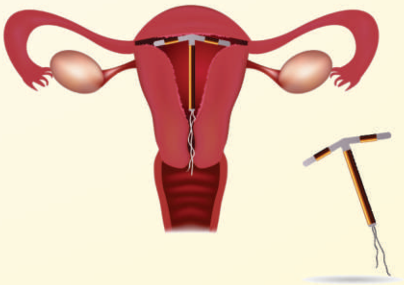

परिवार नियोजन के तरीके

❊ **किशोरी को पोषण एवं स्वास्थ्य सम्बन्धी किन–किन बातों की जानकारी देनी चाहिए?**

आयरन की बड़ी नीली गोली खायें

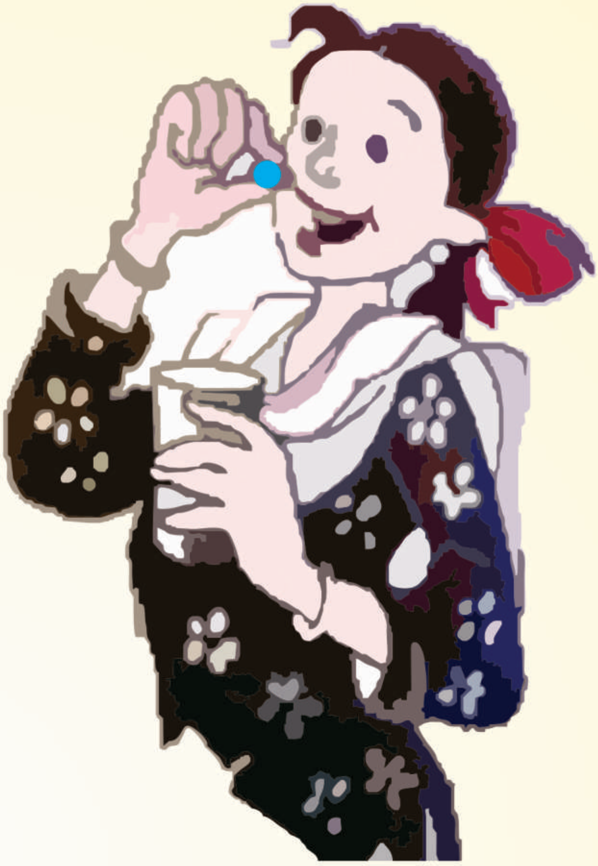

★ संदेश:

- किशोरावस्था 10 से 19 साल के बीच की अवस्था है, किशोरावस्था में शारीरिक विकास तेजी से होता हैं, जिसके कारण किशोरियों को अत्यधिक पोषण की आवश्यकता होती हैं, यदि किशोरावस्था में सही पोषण न मिले और किशोरी कुपोषित रह जाए तो वह भविष्य में कमज़ोर माँ बन सकती हैं।
- किशोरावस्था में एनीमिया से बचाव के लिए एक साल में 52 हफ्तों के लिए WIFS कार्यक्रम के अंतर्गत साप्ताहिक रूप से आयरन फॉलिक एसिड की बड़ी नीली गोली (100 mg एलीमेन्टल आयरन) का सेवन करें।
- कृमि से रोकथाम के लिए साल में दो बार छः–छः माह के अन्तराल पर एल्बेंडाजोल या कृमिनाशक का सेवन करें।
- मासिक धर्म के दौरान साफ –सफाई के लिए प्रोत्साहित करें तथा उससे जुड़ी जानकारियां दे, एवं सेनिट्री नैपकिन के विषय में जानकारी दें। ग्रामीण क्षेत्रों के सभी सरकारी स्कूलों में कक्षा 6 से 12 तक की किशोरियों को निःशुल्क सेनेटरी नेपकिन उपलब्ध कराया जा रहा है।
- किशोरियों को छोटी आयु में विवाह न करना, पोषण, लैंगिक मुद्दे, गर्भ निरोधक, स्वसम्मान, प्रजनन एवं यौन स्वास्थ्य, नशावृत्ति, गैर संचारी रोग इत्यादि बातों की भी जानकारी दें।

मासिक धर्म के दौरान साफ–सफाई पर चर्चा

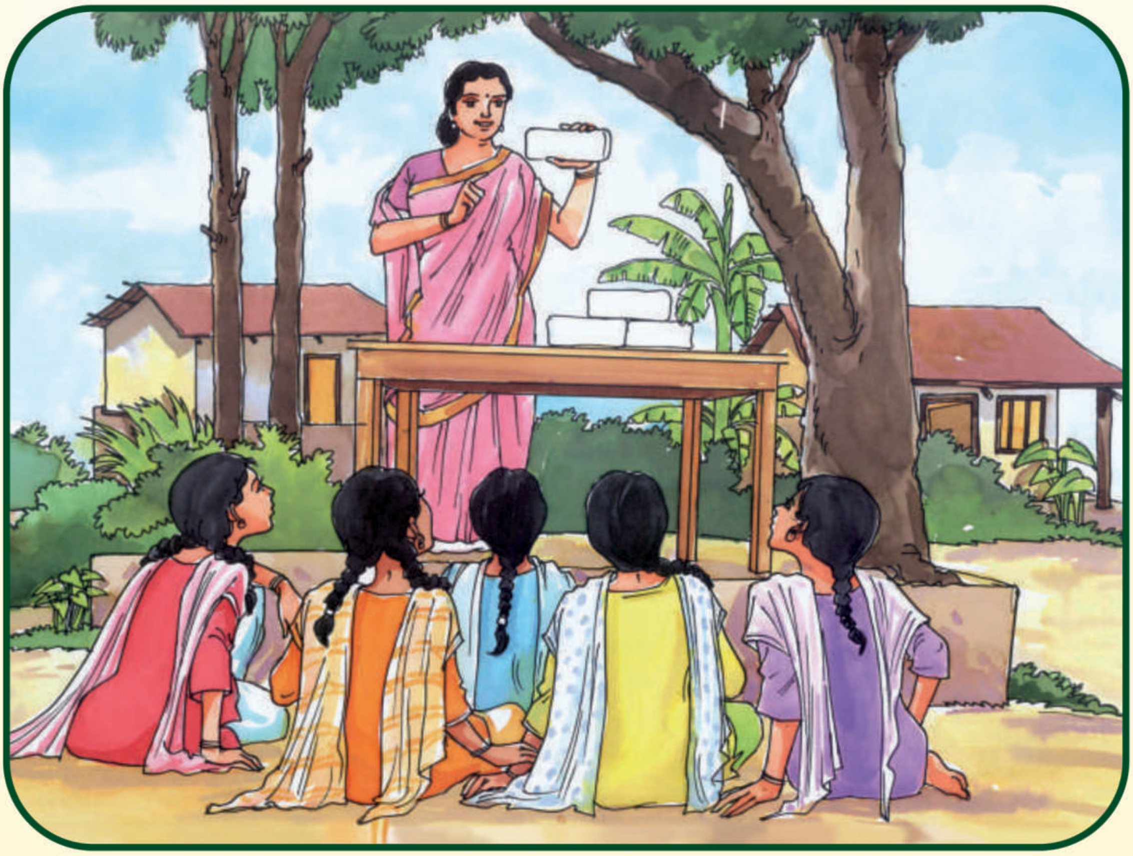

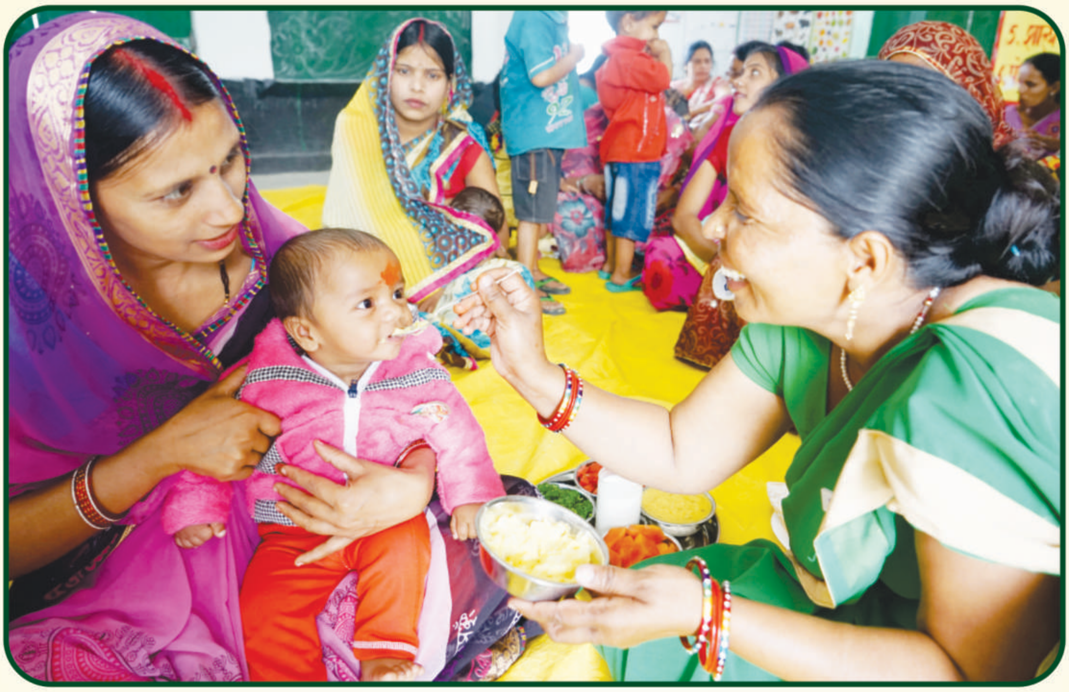

कुपोषण को मात...
आपके हाथ...!
